# Timing of NAD Deficiency During Organogenesis Dictates Defect Type and Penetrance

**DOI:** 10.1096/fj.202502824RRR

**Published:** 2026-02-03

**Authors:** Kayleigh Bozon, Hartmut Cuny, Nana Sunn, Ella M. M. A. Martin, Delicia Z. Sheng, Gavin Chapman, Sally L. Dunwoodie

**Affiliations:** ^1^ Developmental and Stem Cell Division Victor Chang Cardiac Research Institute Sydney NSW Australia; ^2^ University of New South Wales Sydney NSW Australia; ^3^ School of Clinical Medicine, Faculty of Medicine and Health University of New South Wales Sydney NSW Australia; ^4^ Innovation Centre Victor Chang Cardiac Research Institute Sydney NSW Australia

**Keywords:** congenital malformation, embryonic development, magnetic resonance imaging, metabolism, NAD

## Abstract

Major congenital malformations are common, and most cases have no known etiology because of complex interactions between genetic and environmental factors and variable phenotypic outcomes. Congenital NAD Deficiency Disorder (CNDD), a cause of multiple congenital malformations and embryo loss, exemplifies this variability in phenotypic presentation, even between siblings with the same underlying genetic variants. Mouse models show that CNDD is caused by embryonic nicotinamide adenine dinucleotide (NAD) deficiency because of the embryos' genetic inability to synthesize NAD and/or insufficient maternal provision of NAD precursors to embryos. But it is unknown when during pregnancy embryos become susceptible to developing malformations and what drives the malformation variability. Here, we induced CNDD in wild‐type mice via the maternal diet and longitudinally tracked affected and unaffected embryos in utero. We compared 3‐day interval measurements of the maternal blood NAD metabolome with embryo phenotype using Fast Spin Echo Magnetic Resonance Imaging, mass spectrometry, and micro‐computed tomography. Malformations varied between litters, but they correlated with different embryo growth dynamics. Mice with lower maternal NAD Salvage Pathway metabolite levels and minimal levels of derived excretion metabolites from embryonic day 6.5 onward had smaller embryos with more malformations. This showed that altered embryo growth and reduced maternal NAD precursor availability during organogenesis resulted in CNDD. Variability in the timing of maternal metabolic perturbation corresponded to variability in organ and tissue defect types between litters. As embryo phenotypes are directly linked to maternal NAD precursor availability prior to and during organogenesis, this suggests NAD‐derived metabolites are potential biomarkers predicting CNDD.

## Graphical abstract

Pregnant mice on a vitamin B3‐free and tryptophan‐limited diet have embryos with Congenital NAD Deficiency Disorder, characterized by malformations and/or death. We longitudinally tracked the maternal NAD metabolome, embryo growth, and malformation occurrence. Mice with lower NAD Salvage Pathway metabolite levels and minimal levels of derived excretion metabolites from embryonic day 6.5 onward had smaller embryos with more malformations. Thus, embryo phenotypes depend on maternal NAD precursor provision prior to and during organogenesis. *Created in BioRender. Cuny, H. (2025)* https://BioRender.com/m8ct02n.

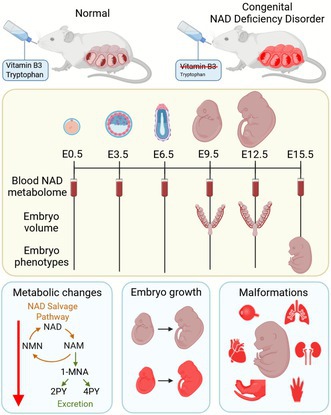

## 1 Introduction

Severe congenital malformations affect up to 3% of live births globally and occur in isolation or in combinations of multiple defects [1]. A major proportion of cases has no known genetic cause because the etiology is often complex, driven by multiple genetic and environmental factors, and gene–environment interactions [2]. The challenge of identifying causative factors of congenital malformation is further complicated by potential heterogeneity in defect types and severity occurring between patients, even with the same genetic predisposition [3].

One such example of a multiple congenital malformation syndrome with variable phenotypic outcome is Congenital NAD Deficiency Disorder (CNDD) [4]. All human patients identified to date have biallelic loss‐of‐function variants in *HAAO*, *KYNU*, or *NADSYN1*, genes encoding enzymes of the NAD *de novo* Synthesis Pathway [5, 6, 7]. CNDD patients have variable phenotypes, even between siblings with the same variant [8]. The CNDD phenotypic spectrum and heterogeneity in defect type and penetrance are mirrored by *Haao*‐, *Kynu*‐, and *Nadsyn1*‐loss‐of‐function mouse models [5, 8].

The vitamin B3 vitamer nicotinamide (NAM) is the main NAD precursor in maternal circulation utilized by all cells to generate NAD via the Salvage Pathway. Tryptophan (TRP) is the second major circulatory NAD precursor, used as the substrate in the NAD *de novo* Synthesis Pathway, primarily by the liver. Recent clinical studies show that perturbed NAD metabolism is linked to various pregnancy and health conditions such as recurrent miscarriage [9], preeclampsia [10], diabetes [11, 12], stroke [13], chronic inflammation [14], and neurological disease [15, 16]. In mouse embryos, NAD *de novo* synthesis exclusively occurs in the extra‐embryonic visceral yolk sac endoderm during early organogenesis, before the embryonic liver takes over this function [17]. Embryos that cannot perform NAD *de novo* synthesis because of biallelic loss‐of‐function variants have sufficient NAD and develop normally as long as sufficient maternal vitamin B3 is provided [5], showing that NAD *de novo* synthesis activity is not required as long as sufficient alternative NAD precursors are available via the maternal circulation. Conversely, embryonic NAD deficiency and CNDD‐like defects are induced in heterozygous or even wild‐type embryos by a combined maternal dietary restriction of the NAD precursors TRP and vitamin B3, thereby affecting both the NAD *de novo* and Salvage Pathways [18]. These and other mouse models have shown that differences in embryo phenotype severity are driven by genetic factors affecting NAD synthesis capability, non‐genetic factors such as maternal diet, and gene–environment interactions [4]. The observed litter‐to‐litter phenotypic heterogeneity among genetically homogeneous mice is primarily caused by differences in maternal NAD precursor provision to her embryos during the organogenesis phase of development [19].

Concordant with the role of NAM as the central circulatory NAD precursor for cells to generate NAD [20], maternal plasma NAM and NAM‐derived excretion metabolite levels strongly correlate with embryo NAD levels, which indicates that embryo NAD levels can be predicted by maternal circulatory levels at any given stage of development [19]. However, mouse models suggest that insufficient maternal NAD precursor provision may only need to occur transiently during the organogenesis phase of embryonic development to induce CNDD and that phenotypic differences may be due to different dynamics of precursor availability and embryonic NAD synthesis throughout embryogenesis [5, 17, 21]. To ascertain how maternal NAD precursor availability corresponds to phenotypic outcome, longitudinal approaches are required that allow all embryos of a litter to be assessed at multiple developmental stages.

Magnetic resonance imaging (MRI) is a powerful technique to delineate structures in living organisms and has proven useful to longitudinally assess live embryos in utero since 1998 when it was performed for the first time in rats [22]. Refined techniques allowed imaging of live murine embryos from embryonic day (E)10.5 onward and even resolution of embryonic structures such as heart, brain, liver, and vasculature in mid‐gestation embryos [23, 24, 25]. Similarly, longitudinal tracking of metabolism using tail vein blood has provided valuable data not achievable with single end‐point measurements [26, 27].

Here, we combined longitudinal tracking of embryo growth with longitudinal measurement of maternal NAD metabolism and embryo phenotype analysis in a CNDD mouse model. This delineated maternal NAD metabolome dynamics during critical stages of embryo development, linked this to individual embryo and whole litter outcomes, and showed that the timing of maternal metabolic changes corresponds to specific malformation phenotypes.

## 2 Materials and Methods

### 2.1 Animal Experiments

All animal experiments were performed in accordance with protocols approved by the Garvan Institute of Medical Research/St Vincent's Animal Experimentation Ethics Committee, Sydney, Australia (approval 21/18). All animal experiments were performed in accordance with the Animal Research: Reporting of In Vivo Experiments (ARRIVE) guidelines and the guidelines and regulations specified in the animal ethics approval. The animal experiments include dietary and embryo phenotypic control groups. Numbers of mice used per experiment is indicated in the respective figures or supplemental tables. No experimental mice were excluded.

Female C57BL/6J wild‐type (WT) mice (RRID:IMSR_JAX:000664) were fed a *Standard Diet* with defined NAD precursor content (Specialty Feeds, Yanderra, NSW, Australia; Cat. #: SF22‐101) for at least 3 weeks prior to mating with a WT male. The presence of a vaginal copulation plug in the morning, defined as timepoint E0.5, confirmed successful mating. From this timepoint onward, pregnant females were either kept on the *Standard Diet* or the feed was replaced with a feed depleted in both vitamin B3 and tryptophan (NAD precursor vitamin‐free and tryptophan‐free feed (ncNTF); Specialty Feeds Cat. #: SF21‐083). Mice given ncNTF were supplemented with 600 mg/L tryptophan in the drinking water (tryptophan‐supplemented water; TW), as described previously [18] and summarized in Table S1. Pregnant females were maintained on their respective diets until dissection at E15.5.

Approximately 15 μL of whole blood was collected from the tail vein of pregnant females, as described previously [28] at E0.5, prior to their transfer to their new diet, and in the morning every 3 days thereafter, up to and including E15.5. Briefly, blood was collected from the tail of restrained mice using an EDTA‐coated 20 μL Minivette Point‐of‐Care Test device (Sarstedt, Nümbrecht, Germany; Cat. #: 17.2113.020). Whole blood was immediately transferred to 1.5 mL microcentrifugation tubes and snap frozen for storage until metabolomic analysis. In utero MRI was performed after blood collections at E6.5, E9.5, and E12.5.

At E15.5, pregnant females were sacrificed by cervical dislocation and embryos dissected in ice‐cold phosphate‐buffered saline, with the position of live and dead embryos within the uterine horn noted. External phenotyping was performed using light microscopy, including for structural malformations of the neural tube (exencephaly), eyes, tail (caudal agenesis), palate, limbs, digits, and presence of oedema. Control females provided the *Standard Diet* throughout, but without exposure to MR imaging, or tail vein blood collection were also collected at E15.5 to establish normal organ volume thresholds for the automated phenotyping platform (see Supporting Information Methods for details).

### 2.2 Fast Spin Echo Magnetic Resonance Imaging (FSE‐MRI)

In utero MRI scans were performed on pregnant mice (*n* = 7) at gestational stages E6.5, E9.5 and E12.5 using a horizontal 7 T PET/MRI (Dry Magnet Model 7024, MR Solutions, Guildford, UK). The radio frequency coil used was a mouse body coil (38 mm diameter, quadrature bird cage, MR Solutions). As embryos could not always be accurately identified at E6.5, all remaining mice (*n* = 13) were imaged at E9.5 and E12.5 only. Briefly, anesthesia was induced using 3% isoflurane, which was reduced to 1.3%–1.8% during the MRI scan. Each mouse was placed in the mouse bed (Minerve, France) in a supine position and kept warm with an in‐built heated airflow system at 36°C. The animal's physiology parameters (heart rate, temperature, respiration) were monitored using PC‐SAM (Model 1030, SAII, USA). Images were acquired in the coronal plane using a field of view of 40 mm × 40 mm. Thirty 0.5 mm thick slices were acquired to cover the entire abdominal cavity containing the litter of embryos. T2‐weighted images were acquired using the Fast Spin Echo (FSE) method with TR = 4000 ms, TE = 45 ms, averages = 8, Echo Train = 7, Matrix = 256 × 196 and total scan time = ~15 min.

### 2.3 Segmentation of in Utero MR Images in Amira

After an MRI was performed, Digital Imaging and Communications in Medicine (DICOM) images were loaded in Amira3D v2022.1 (RRID:SCR_007353). Voxel size of each scan was 0.2041 × 0.5 × 0.1563 mm. Each embryo was segmented in the segmentation editor, as described previously [29] using the yolk sac as a boundary, and tracked across three stages. Three‐dimensional volumes were extracted using the material statistics module.

### 2.4 Micro‐Computed Tomography (Micro‐CT) Imaging and Phenotypic Analyses of Embryos

After external phenotyping, E15.5 embryos were rinsed in ice‐cold distilled water, then transferred into ice‐cold phosphotungstic acid (PTA) staining solution (0.7% PTA/70% ethanol) and placed on a tube roller at room temperature for up to 3 weeks, with PTA/ethanol solution replaced once a week. Micro‐CT scanning was performed as described previously [8] with minor adaptations. Briefly, once mounted in 1% agarose gel, embryos were imaged using a Skyscan 1272 high‐resolution 3D X‐ray microscope (Bruker, Billerica, MA, USA) operating at a 75 kV source voltage, 133 μA source current, and an Al 0.5 + Cu 0.038 mm filter. Magnification was set at a pixel size of 6 μm^3^ and images taken at 0.4° increments through 360°, with two fields of view and 2500 ms exposure per image. NRecon software (Bruker) was used to reconstruct micro‐CT acquired projections into cross‐section images with smoothing (9), ring artifact (19), and post‐alignment corrections. Reconstructed datasets for each embryo were cropped and down sampled to 14 μm^3^ in HARP software (Harwell Automated Reconstruction Processor, v2.3.2; RRID:SCR_016546) and saved in nearly raw raster data (NRRD) format. All embryos were assessed manually as follows: NRRD files were loaded into ITKSnap (RRID:SCR_002010) to confirm external phenotypes identified at dissection and identify morphological defects in internal organs, including the heart, lungs, and kidneys.

To quantify the severity of embryo outcomes, the number of malformed organs/tissues was counted. Embryos with a single affected organ/tissue and those with multiple affected organs/tissues were classified as having isolated and multiple defects, respectively. Defect types included in analyses were exencephaly, anophthalmia, microphthalmia, cleft palate, short or missing limbs, digit defects (missing, extra, and/or fused digits), caudal agenesis, oedema, structural heart defects, kidney hypoplasia or agenesis, abnormal lung lobes, underdeveloped lung parenchyma, and lung hypoplasia.

Kidney and lung volumes were determined using the automated image analysis pipeline LAMA (RRID:SCR_019133) [30]. Individual embryos were registered to a PTA‐based E15.5 population average, and organ volumes were calculated by reverse registration to an existing E15.5 organ atlas [31]. Lung and kidney hypoplasia were identified using adapted organ labels from the existing E15.5 atlas [31] in which four existing right lung lobe labels (cranial, middle, caudal, and accessory lung label) were combined to form one right lung label. The resulting segmentations were amended where necessary using Amira, and LAMA permutation statistics were performed comparing the control with the treated groups. Lung (left or right) or kidney (left or right) were deemed hypoplastic if both of the following criteria were met: the organ was significantly different when that embryo's organ volume was compared to the baseline group (embryos on *Standard Diet* without isoflurane treatment) following false discovery rate (FDR) adjustment to 0.2, and the percentage change in normalized organ volume was > 3 standard deviations from the baseline average.

Additional automated analysis was performed to assess the effect of 2× and 3× isoflurane treatment. Organ volumes were determined and adjusted as described above. Embryos from 2× and 3× isoflurane treatment groups were separately compared to embryos on the *Standard Diet* that were neither isoflurane‐treated nor MRI‐imaged. To be classified as different in organ volume, results had to meet the following criteria: the organ was significantly different between the treatment and controls in permutation statistics (FDR‐corrected to 0.01) and standard Benjamini‐Hochberg correction [32] (corrected *p* value < 0.01), and the volume difference was large, defined as Cohens d > 0.8 or < −0.8 [33, 34].

### 2.5 Metabolite Quantification in Whole Blood

NAD‐related metabolites tryptophan (TRP), kynurenine (KYN), kynurenic acid (KA), NAD^+^, nicotinamide (NAM), nicotinamide mononucleotide (NMN), 1‐methylnicotinamide (1MNA), N‐methyl‐2‐pyridone‐5‐carboxamide (2PY), and N‐methyl‐4‐pyridone‐3‐carboxamide (4PY) were quantified in tail vein whole blood samples by ultra‐high performance liquid chromatography—tandem mass spectrometry (UHPLC–MS/MS), as described previously [35] with minor adjustments to the metabolite extraction procedure to account for the smaller sample volumes. Briefly, blood (10 μL) was diluted in 90 μL water, then processed as described except that samples were not sonicated. Pilot experiments determined that the NAD‐related metabolites nicotinic acid, nicotinic acid adenine dinucleotide, nicotinic acid mononucleotide, 3‐hydroxykynurenine, anthranilic acid, and xanthurenic acid were below the detection limit because of very low levels in murine blood and/or did not consistently meet the UHPLC–MS/MS quality control parameters. These were excluded from analysis.

### 2.6 Statistical Analysis

All one‐way ANOVA, *t*‐test, and Spearman's correlation analyses of embryo volume data were performed using GraphPad Prism (version 10; RRID:SCR_002798). Multiple Correspondence Analysis (MCA) analyses were performed in R (RRID:SCR_001905) using FactoMineR [36] (RRID:SCR_014602) default settings for embryos of the *Limited Diet* only. For these analyses, main organ/tissue defects or their subtypes were only included if their incidence was over five across all embryos assessed.

Linear mixed model analyses to compare different numbers of isoflurane treatments were performed in RStudio (RRID:SCR_000432) using lme4 [37] for the following equation: “volume ~ time × number of isoflurane treatments × dietary treatment + (1 | embryo)”. Within this model, time, number of isoflurane treatments, and dietary treatments were fixed effects, and random slopes were generated per individual embryo. The linear mixed model was plotted with flexplot [38].

MetaboAnalyst 6.0 [39] (RRID:SCR_015539) was used for the analysis of whole blood NAD metabolome data except for Kruskal‐Wallis one‐way ANOVA, for which GraphPad Prism was used. In metabolite scatter plots, metabolite levels below the limit of detection (LOD) were given a value LOD/2 to allow statistical comparisons. To allow comparisons across metabolites with different concentration ranges in MetaboAnalyst, measured concentrations were standardized by log10 transformation and Pareto scaling. Standardized concentration data were used to generate heatmaps, Principal Component Analysis (PCA) plots, and Partial Least Squares Discriminant Analysis (PLS‐DA) plots.

## 3 Results

### 3.1 MRI‐Mediated Longitudinal Analysis Shows Consistent Growth Trajectories Among Embryos Without Malformation

**FIGURE 1:**
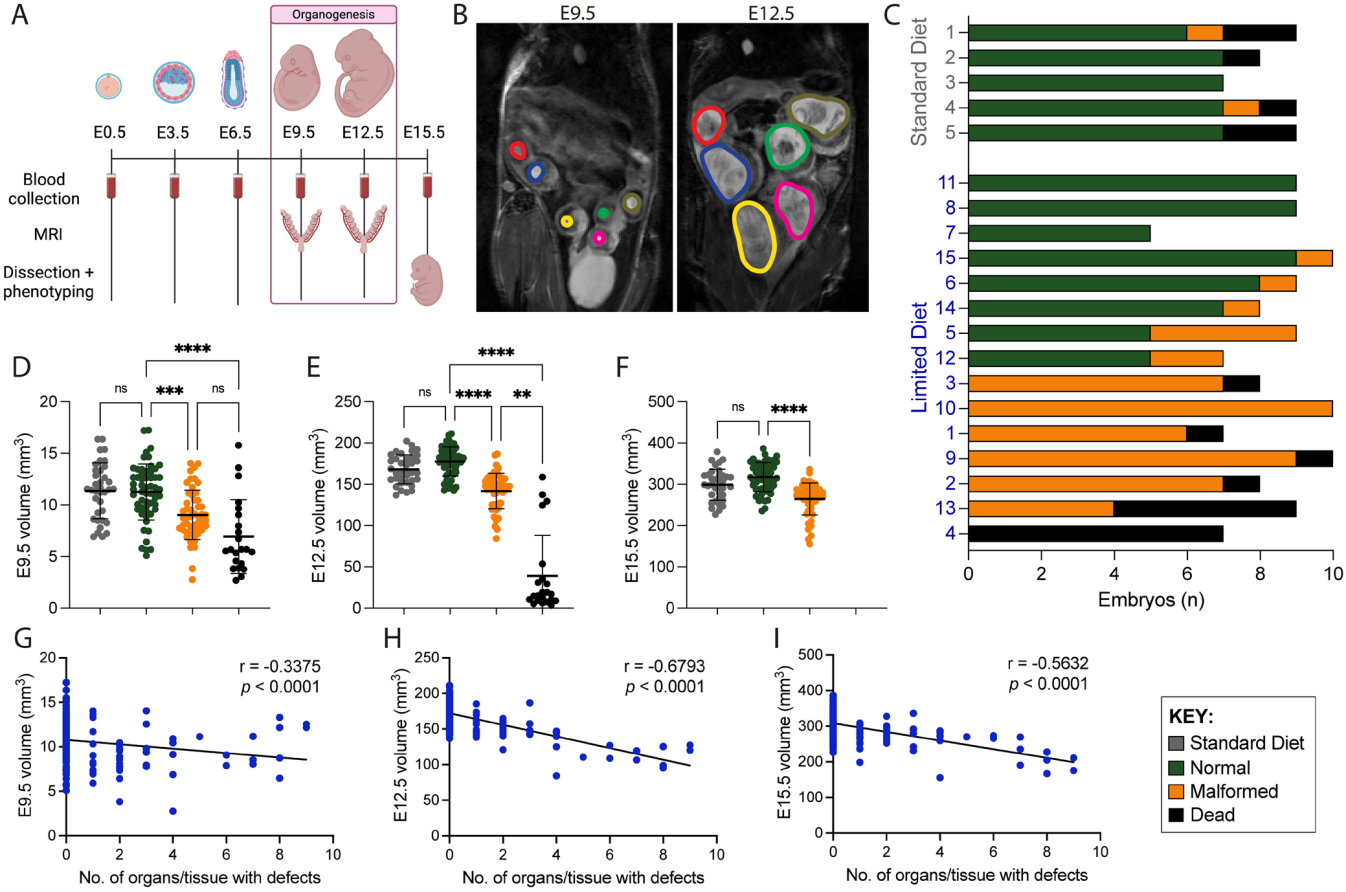
Longitudinal analyses of murine embryos in utero. (A) Schematic overview of the experimental procedures. Tail vein blood was collected from pregnant females every three days from E0.5 to E15.5. MRI scans were performed at E9.5 and E12.5. Embryos were dissected and phenotyped at E15.5. The approximate phase of embryo organogenesis is indicated. (B) Representative MRI images taken at E9.5 and E12.5. Embryos are outlined by colored lines. (C) Summary of phenotypic embryo outcomes at E15.5 induced by different maternal diets. Each bar represents a litter. Colors indicate whether an embryo was phenotypically normal (green), malformed (orange) or dead (black). The diets given to female mice throughout pregnancy and the litter number are indicated on the left. For diet specifics, see Table S1. Embryos classified as normal had no malformations whereas those classified as malformed had one or more affected organs/tissues. See Methods for included defect types. Dead embryos were defined as being resorbed by E15.5. (D–F) Embryo volumes measured by MRI at E9.5 (D), E12.5 (E), and E15.5 (F). Each dot represents an embryo, and bars indicate mean ± standard deviation. Embryos were grouped according to the maternal diet (*Standard Diet*: Gray, *Limited Diet*: Green, orange, black). Embryos on the *Limited Diet* were separated according to their phenotype, with the same categories as in (C). Significance of difference between groups was assessed by Kruskal‐Wallis one‐way ANOVA with Dunn's multiple comparisons test (****p < 0.0001, ***p < 0.001, **p < 0.01; ns, not significant). (G–I) Graphs plotting the correlation between embryo volumes and the numbers of organs/tissues with defects in the respective embryo at E9.5 (G), E12.5 (H), and E15.5 (I). The line indicates simple linear regression. Significance of relationship between embryo volume and the number of organs/tissues with defects was calculated using Spearman's Correlation (*r*).

We tracked embryos from five WT mothers on *Standard Diet* via FSE‐MRI to determine their general growth dynamics and collected them at E15.5 for phenotyping (Figure 1A). Successful tracking and volumetric measurement of all embryos (live and dead) were possible from E9.5 onward (Figures 1B, S1 and S2, Supporting Information Dataset 1), but not all embryos were reliably identified at E6.5 during preliminary experiments.

To test whether isoflurane treatment for the MRI scan at E6.5 negatively impacted embryo development relative to embryos scanned at E9.5 and E12.5 only, we compared embryo volumes at E9.5 and E12.5 and phenotypes at E15.5 between the 2× and 3× isoflurane treatment groups. The 3× treated embryos were significantly smaller (Figure S3A–C). Linear regression analysis of embryo volume over time showed that differences in embryo volume/size were not due to altered growth dynamics (Figure S3D,E) but because of a smaller size at E9.5 (Figure S3A). To determine potential effects on embryo malformation phenotypes, we used an automated analysis pipeline, LAMA [30, 31], and compared organ volumes between embryos of the 2× and 3× treatment groups and embryos on the same diet but not exposed to isoflurane. This was done for all organs in the E15.5 organ atlas (Supporting Information Dataset 2). Of the 43 organ labels assessed, only four exhibited significant differences according to the set criteria: left lung, spinal cord, submandibular glands, and trachea (Supporting Information Dataset 2). Observed changes in organ volume relative to the control group were consistent between the two isoflurane‐treated groups, but the effect was generally larger for the 2× isoflurane‐treated embryos. Consequently, left lung and submandibular gland increased in size relative to non‐isoflurane‐treated controls were only significant in the 2× treated group.

In summary, the additional isoflurane treatment at E6.5 affected embryo size but did not affect embryo phenotype. Observed differences in organ volumes relative to a non‐treated control group were consistent between 2× and 3× treated embryos. Therefore, data from 2× and 3× treatment were combined for subsequent analyses.

Next, we determined how maternal NAD precursor restriction during pregnancy affects embryo phenotype and growth dynamics. NAD precursor restricted *Limited Diet* (Table S1) was given to 15 pregnant WT mice from confirmation of pregnancy (E0.5) until they were sacrificed and embryos collected at E15.5. The TRP content in the *Limited Diet* is the same as in diets used to induce CNDD in previous studies. These showed that it is the lack of sufficient precursors to synthesize NAD, irrespective of whether this is TRP or vitamin B3, and not a lack of TRP for other biosynthetic processes, that correlates with malformation occurrence because TRP limitation, as in *Limited Diet*, does not cause malformations when compensated by additional vitamin B3 [18, 19, 21]. Such diets are required because mice are very efficient in generating NAD via the Salvage Pathway, and CNDD can only be induced in wild‐type mice/embryos when Salvage Pathway precursors are essentially derived from TRP and TRP provision is also lowered [17, 18].

Phenotypic analysis showed that 47.7% of embryos (52/109) had defects consistent with CNDD. There was significant phenotypic variation, ranging from litters where all embryos were entirely normal to those in which all embryos were either malformed or dead (Figure 1C; see Supporting Information Dataset 1 for detailed phenotyping data), as observed in similar models [8, 18, 19, 21]. Entire litters were tracked from mid‐gestation onward via FSE‐MRI, thereby allowing investigation into how embryo growth dynamics relate to phenotypic outcomes.

Malformed embryos were on average smaller than embryos without malformations at all three stages assessed (Figure 1D–F, Table S2). To determine whether the *Limited Diet* affected embryonic growth dynamics generally, because of a general nutritional deficiency of, for example, TRP, independent of malformation, we compared volumes of embryos without malformations from the *Standard Diet* and *Limited Diet* groups. There was no difference in embryo volume at any stage (Figure 1D–F, Table S2). This was further confirmed by linear mixed model analyses which demonstrated that *Limited Diet* had no effect on the growth dynamics of embryos without malformations (*p* = 0.64689) (Figure S3F). This indicated that the reduced growth phenotype seen in malformed litters is linked to malformation occurrence.

### 3.2 Embryo Size in Utero Correlates With Malformation Load and Embryo Survival

The number of isoflurane treatments did not affect the volume of malformed embryos from mothers on *Limited Diet* (Figure S3G–I). Instead, the more defects an embryo had at E15.5, the more its growth was impacted in utero, as evidenced by the significant negative correlation between embryo volume and number of defects, which was subtle at E9.5 but obvious at E12.5 and E15.5 (Figure 1G–I). Accordingly, embryo volumes varied between litters at all three stages, and litters with all embryos affected had the lowest average embryo volumes (Figure S4).

Similar to previous CNDD models [5, 8, 18, 19, 21], there was an elevated rate of in utero death (12.8%; 16/125) in litters on *Limited Diet*. Longitudinal MRI scanning successfully tracked these embryos, because all embryos identified as dead at E15.5 had corresponding implantation sites at both E9.5 (Figure S1) and E12.5 (Figure S2). Two distinct categories of death were identified at E15.5: early embryo loss, defined by the lack of embryonic tissue remnants (12/16, 75%), and late embryo loss, defined by the presence of necrotic embryonic tissue (4/16, 25%) at E15.5. Dead embryos were, on average, significantly smaller at E9.5 than normal embryos (Figure 1D, Table S2). At E12.5, early resorptions were distinguishable by both their significantly smaller size (Figure 1E, Table S2) and reduced brightness in MRI images. By contrast, those four embryos representing late embryo loss were of similar size and appearance at E9.5 and E12.5 as surviving malformed embryos (Figure 1E), suggesting they died between E12.5 and E15.5. Therefore, longitudinal tracking of embryos characterized differences in both embryo size and growth relative to their malformation load and identified which embryos died in utero prior to dissection at E15.5.

### 3.3 Co‐Occurrence of Defect Subtypes Follows Distinct Patterns That Are Conserved Within Litters

**FIGURE 2:**
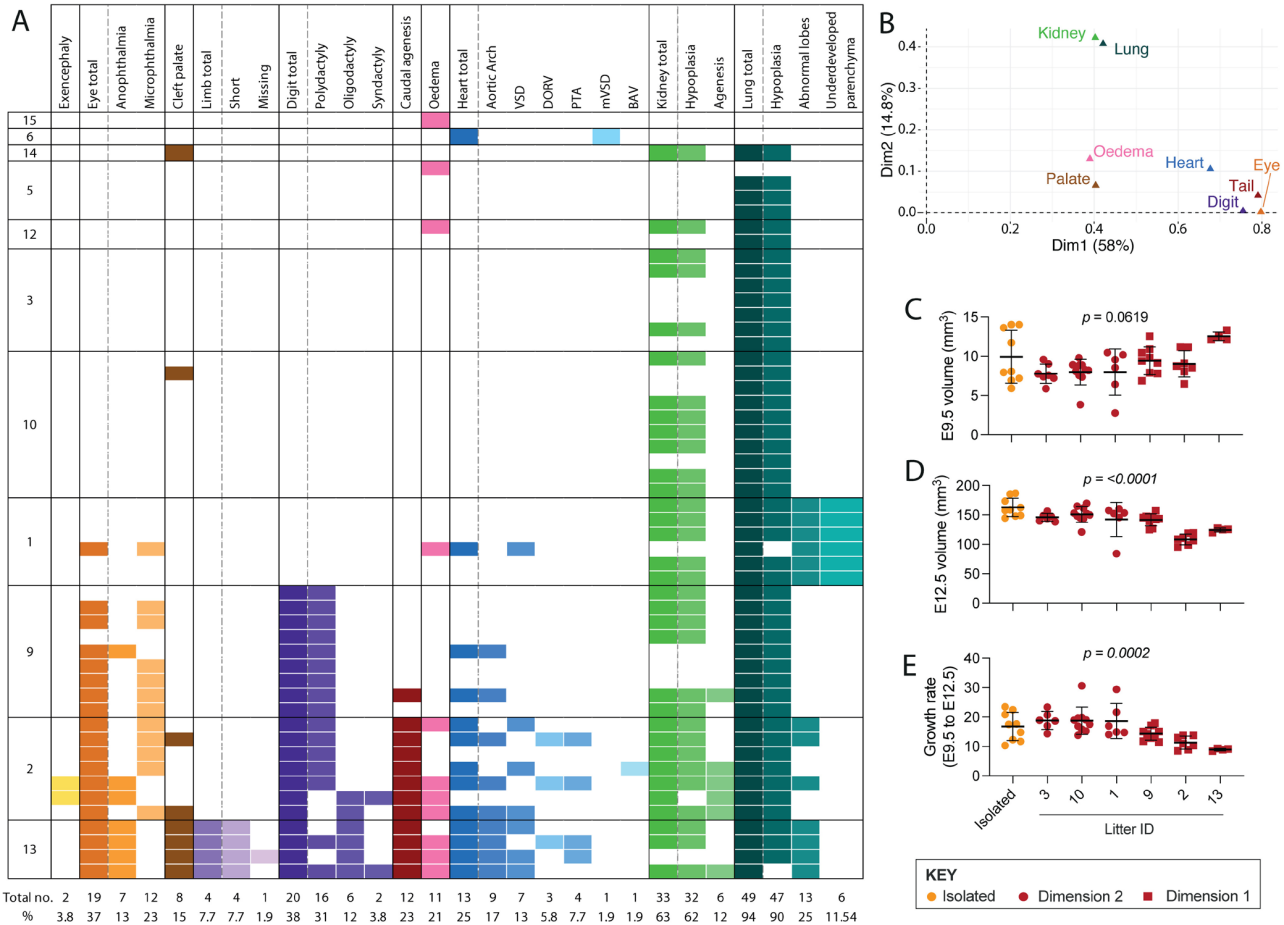
Embryo malformations at E15.5 show patterns of co‐occurrence. (A) Summary of embryo defect types. Each row represents an embryo, and identified defect types are highlighted in color. Embryos are sorted by litters, with the litter ID indicated in the leftmost column. The total number of embryos and percentage of embryos among all embryos with defects are indicated at the bottom. (B) Multiple correspondence analysis (MCA) to assess co‐occurrences of defects in main organ/tissue categories. Colors match those in (A) to indicate the corresponding organ/tissue. Organ/tissue categories were removed from analyses if there were insufficient incidences (*n* < 5) among *Limited Diet* embryos. (C–E) Comparison of embryo volumes at E9.5 (C), E12.5 (D), and the growth rate from E9.5 to E12.5 (E) between litters with defects in < 1 embryo per litter (litters 15, 6, 14, 5, and 12; grouped and categorized as Isolated) and those litters with an average of > 1 defect per embryo (litters 3, 10, 1, 8, 2, 13; each litter shown individually). Litters 3, 10, and 1 align with MCA Dimension 2, whilst litters 9, 2, and 13 align with MCA Dimension 1. Each dot represents an embryo, and bars indicate mean ± standard deviation. Significance of difference between groups was assessed by Kruskal‐Wallis one‐way ANOVA, with *p* values indicated above graphs.

Malformations in embryos on the *Limited Diet* varied, ranging from isolated defects to co‐occurring defects in up to nine different organs/tissues (Figure 2A, Supporting Information Dataset 1). Furthermore, some embryos had more than one malformation per affected organ/tissue, such as the co‐occurrence of two heart defects (Figure 2A, Supporting Information Dataset 1). The lung was the most frequently affected organ, with malformations (predominantly hypoplasia) present in 45% of all live embryos and 94.2% of affected embryos (49/52), on the *Limited Diet* (Figure 2A). Other malformations tended to co‐occur with lung defects, except for heart defects (one embryo) or oedema (two embryos), which were also observed in isolation (Figure 2A). Kidney defects (hypoplasia or agenesis) were the next most common, present in 63.5% of malformed embryos (33/52) (Figure 2A). Other defects affected a smaller subset of embryos generally and tended to cluster within litters (Figure 2A).

To explore the relationships between different defects, MCA were performed for the main organ categories for all embryos on the *Limited Die*t. The first two dimensions explained the majority (72.8%) of the observed variance in defect formation (Figure 2B). All organs/tissues correlated with Dimension 1 (58%), but the grouping of eye, digit, tail, and heart at the positive extreme end of Dimension 1 (Figure 2B) illustrated their close association with one another. Indeed, these defects were concentrated almost exclusively in *Limited Diet* litters #9, #2, and #13 (Figure 2A). By contrast, the strong association of lung and kidney malformation with Dimension 2 (Figure 2B) reflects their frequent co‐occurrence, as observed in 63.5% of all embryos. They frequently co‐occurred without any other defects, and this was most prevalent in litters #12, #3, #10, and #1 (Figure 2A).

Additional MCA of malformation subsets (Figure S5A) provided more refined insights into these dimensions. Although all heart defect subtypes were associated with Dimension 1, lung, kidney, eye and digit subtypes separated across the two dimensions. Dimension 2 was primarily driven by lung hypoplasia and kidney hypoplasia (Figure S5A), a co‐occurring phenotype observed in most embryos of litters #5, #12 and #10 (Figure 2A). Other lung and kidney defect subtypes were either related to Dimension 2 (underdeveloped lung parenchyma) or strongly associated with Dimension 1 (abnormal lung lobes or kidney agenesis). Therefore, the two MCA dimensions represent distinct embryonic outcomes and reflect inter‐litter phenotypic variability.

Given this variability, embryo volume and growth rate of malformed embryos were compared between litters to determine if the type of defect differentially affected in utero growth. For this analysis, litters where the average number of defects per embryo was < 1 (#15, #6, #14, #5 and #12) were combined into a single group (*Isolated*). There was no significant difference between embryo volume at E9.5 between litters, with embryos that would develop the greatest number of defects (litter #13) having the largest volume at E9.5 (Figure 2C). However, there was a significant difference in growth rate from E9.5 to E12.5 between litters (*p* = 0.0002), which resulted in significant differences in embryo volume by E12.5 (*p* < 0.0001) (Figure 2D,E). Growth rate within this critical window of embryonic development was lower in litters associated with Dimension 1 (#9, #2 and #13; Figure 2E) and declined as the number of organs affected increased (Figure 2A). The significant difference in embryo volume between litters remained at E15.5 (Figure S5B) because there was no significant difference in litter growth rate between E12.5 and E15.5 (Figure S5C).

### 3.4 The Maternal NAD Metabolome Dynamically Changes Over Gestation and Is Affected by Diet

**FIGURE 3:**
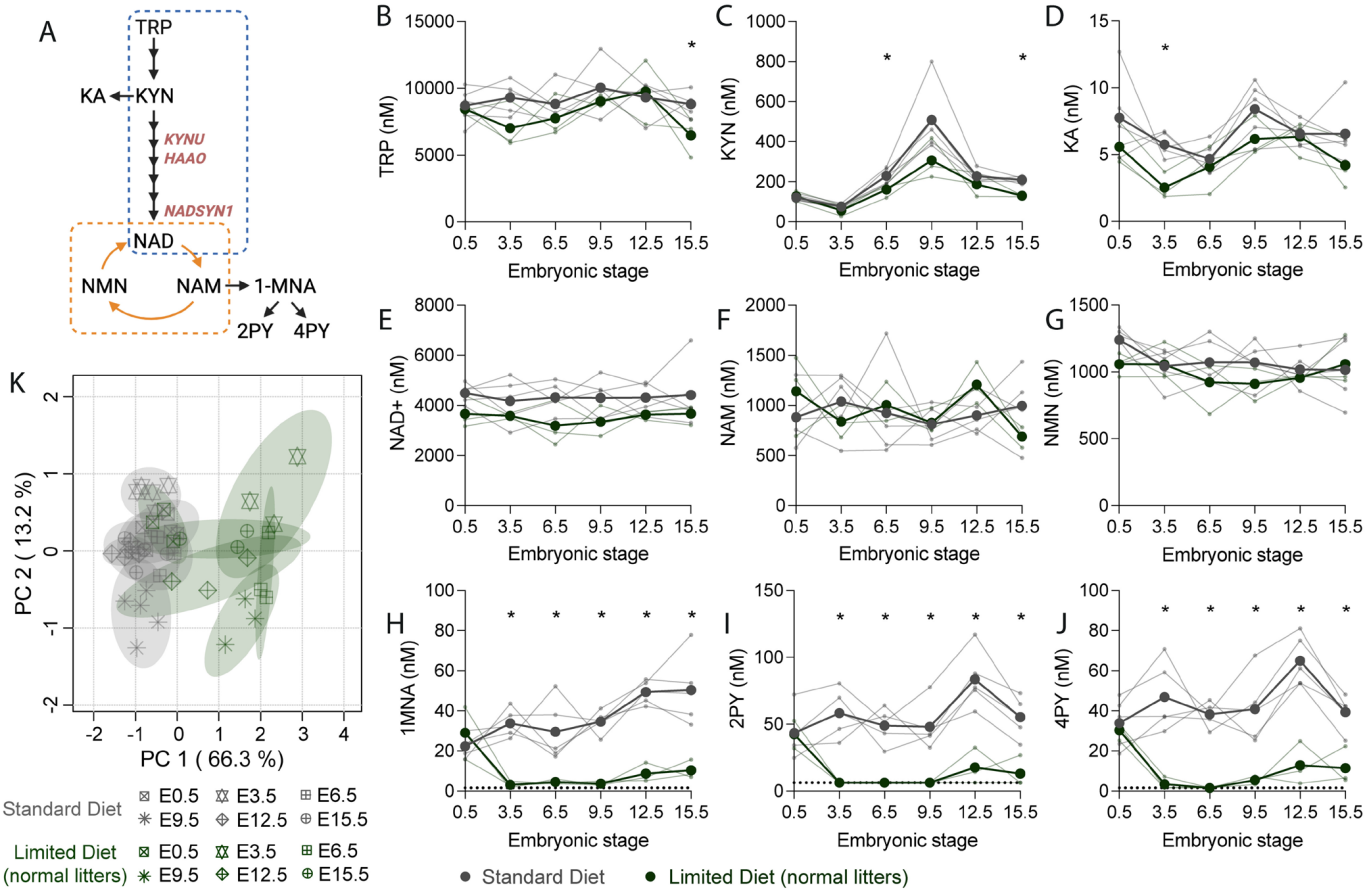
The maternal NAD metabolome is dynamic across gestation and is influenced by dietary NAD precursor provision. (A) Overview of the NAD synthesis pathways. The metabolites monitored in this study are highlighted. The NAD *de novo* Synthesis Pathway is outlined by a blue dashed line and the NAD Salvage Pathway by an orange dashed line. (B–J) Metabolite levels measured longitudinally from E0.5 to E15.5 in whole blood of mothers on the *Standard Diet* (gray) and mothers on *Limited Diet* with litters consisting of entirely normal embryos (green), whereby normal phenotype refers to the absence of malformation. Large dots and bold lines indicate the average values and small dots and thin lines the values of individual mice. The detection limits of 1MNA, 2PY, and 4PY are outlined by dotted lines. Significance of difference between groups at each timepoint was assessed by unpaired *t*‐test, with *p < 0.05 indicated above the graphs where applicable. Additional statistical tests to assess differences in metabolite levels within the same group but across time are reported in Table S3. (K) Principal Component Analysis of metabolite levels including both diets and all timepoints as in (B–J). Concentration values were standardized (log10 transformed and Pareto scaled) for analysis. Principal component 1 (PC1) shows a separation by diet, whereas PC2 represents the separation by time. KA, kynurenic acid; KYN, kynurenine; NAD^+^, nicotinamide adenine dinucleotide (oxidized); NAM, nicotinamide; NMN, nicotinamide mononucleotide; TRP, tryptophan; 1MNA, 1‐methylnicotinamide; 2PY, N‐methyl‐2‐pyridone‐5‐carboxamide; 4PY, N‐methyl‐4‐pyridone‐3‐carboxamide.

Previous studies suggest that the maternal NAD metabolome is dynamic during pregnancy [19, 21], but this is inferred from different females collected at different endpoints. Therefore, we next determined how the levels of nine NAD‐related metabolites (TRP, KYN, KA, NAD^+^, NAM, NMN, 1MNA, 2PY, and 4PY; Figure 3A) changed in the maternal blood of the same mouse throughout pregnancy. Whole blood NAD metabolite levels are considered a good indicator for tissue NAD metabolite levels and NAD precursor availability [40, 41, 42].

When provided the non‐restricted *Standard Diet*, pregnant mice show dynamic changes in their NAD metabolome. Though the levels of the major NAD precursors TRP, NAM, and NMN, as well as NAD^+^ remained relatively stable over time (Figure 3B,E–G, Table S3; see Supporting Information Dataset 1 for metabolite levels per mouse), the blood concentrations of all NAD Salvage Pathway excretion metabolites (1MNA, 2PY, and 4PY) significantly changed over gestation (*p* ≤ 0.024; Figure 3H–J, Table S3). They all increased between E9.5 and E12.5, then 2PY and 4PY declined between E12.5 and E15.5, as previously observed in mouse plasma [19] (Figure 3I,J; Table S3). KYN levels showed significant dynamic changes across gestation among females on *Standard Diet* (*p* = 0.004), whereby its levels dipped at E3.5 and then increased with a peak at E9.5 before declining by E12.5 and remaining steady between E12.5 and E15.5 (Figure 3C, Table S3). KA, another metabolite of the NAD *de novo* Synthesis Pathway, followed the same trend (Figure 3D, Table S3).

Maternal dietary NAD precursor provision, even when all embryos were phenotypically normal, had a profound effect on NAD metabolism, as demonstrated by the separation of the NAD metabolome by diet on a PCA plot, accounting for 66.3% of the variability along principal component 1 (Figure 3K). Yet, despite the reduction in TRP availability in the *Limited Diet*, blood TRP levels were stable over gestation and not different from those in females provided the *Standard Diet*, except for a significant drop at E15.5 (*p* = 0.0357; Figure 3B, Table S3). Similarly, NAD^+^ levels remained stable throughout gestation, with no significant difference between dietary treatments (Figure 3E, Table S3). By contrast, all NAD Salvage Pathway excretion products (1MNA, 2PY, and 4PY) declined strongly from E0.5 to E3.5, and levels were significantly lower at all stages from E3.5 onward relative to those with *Standard Diet* (Figure 3H–J, Table S3). This reflects previous observations, where the levels of these metabolites declined in mice under conditions of decreased dietary NAD precursor availability [8]. KYN levels exhibited the same dynamic changes as seen with the *Standard Diet*, but to a lesser extent (Figure 3C, Table S3).

### 3.5 Malformation Load Is Inversely Correlated With NAD ^+^ and Excretion Metabolite Levels in Maternal Blood

**FIGURE 4:**
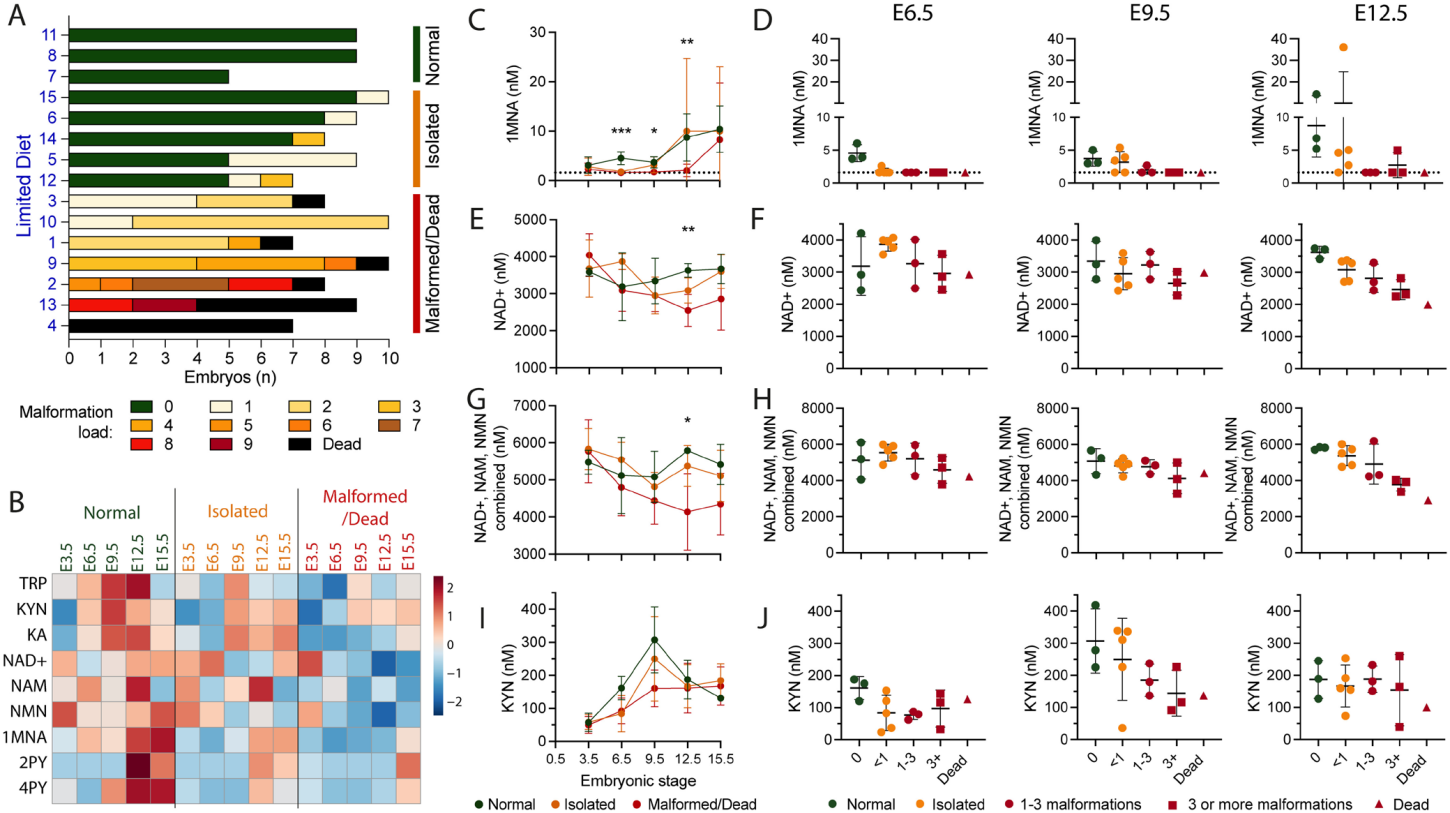
Pregnant mice with affected litters had NAD metabolomic differences compared to females with unaffected litters. (A) Summary of phenotypic embryo outcomes at E15.5 indicating the malformation load (number of affected organs/tissues) of embryos. Each bar represents a litter, and each embryo is represented by a color according to how many organs/tissues were malformed, or whether the embryo died in utero. Litters were sorted by malformation load from entirely unaffected to entirely dead and categorized into three groups: *Normal* (none of the embryos of the litter had malformations), *Isolated* (average number of affected organs/tissues < 1 per embryo), and *Malformed/Dead* (average number of affected organs/tissues > 1 per embryo or embryo dead). (B) Heatmap showing standardized (log10 transformed and Pareto scaled) metabolite concentrations of pregnant females on the *Limited Diet* from E3.5 to E15.5. Litters were grouped into each category as outlined in (A). (C, E, G, I) Metabolite levels longitudinally measured from E3.5 to E15.5 in whole blood of mothers on the *Limited Diet*. Colors indicate the categories on the basis of litter malformation load, as outlined in (A). The detection limit of 1MNA is indicated by a dotted line. Significantly different values between the groups at a given timepoint, determined by Kruskal‐Wallis one‐way ANOVA are shown (***p < 0.001, **p < 0.01, *p < 0.05). See Table S7 for numerical *p* values. (D, F, H, J) Scatter plots of the values at E6.5, E9.5, and E12.5 of the respective metabolites in (C, E, G, I). Each dot represents a blood sample, and bars indicate mean ± standard deviation. The Malformed/Dead group was stratified into three subgroups to highlight trends between malformation load and metabolite levels: average number of affected organs/tissues 1–3 (circles), > 3 (squares), or all embryos dead (triangles). KYN, kynurenine; NAD^+^, nicotinamide adenine dinucleotide (oxidized); NAM, nicotinamide; NMN, nicotinamide mononucleotide; 1MNA, 1‐methylnicotinamide.

Next, we assessed how the NAD metabolome of pregnant females on *Limited Diet* correlated to litter outcomes. Given the heterogeneity in embryo phenotypes, pregnant females were categorized into three groups according to the average number of affected organs/tissues per embryo for the whole litter. The *Normal* category included litters without malformed embryos, the *Isolated* group included litters with some malformed embryos and an average of < 1 affected organs/tissues per embryo, and the *Malformed/Dead* group included those in which all embryos were affected, either with an average number of > 1 affected organs/tissues per embryo or being dead (Figure 4A).

Levels of seven of the nine metabolites were highly variable among mice in the first maternal tail vein blood sample (E0.5) taken at the timepoint of diet change to *Limited Diet*, with a coefficient of variation of > 15%. However, none of the nine metabolites had significantly different levels between the litter phenotype groupings (Table S4, Figure S6A–I), suggesting that metabolite levels at E0.5 showed some inherent variability, independent of the phenotypic litter outcome later in gestation. This is supported by PCA and PLS‐DA analyses, which both showed no separation between any of the groups *Standard Diet*, *Normal*, *Isolated*, or *Malformed/Dead* (Figure S6J–K).

In blood samples taken between E3.5 and E15.5, both the absolute concentrations as well as the concentration dynamics across gestation varied between litter phenotype categories for most metabolites (Figure 4B, Table S5). TRP levels were generally variable over time and between mice, but they trended slightly lower in mice with affected litters (Figure S7G,H). Levels of the NAD *de novo* Synthesis Pathway metabolites KYN and KA generally increased from E3.5 onward (Figure 4I, Figure S7I). Although mothers with normal litters showed the same peak in both metabolites at E9.5 as mothers on *Standard Diet* (Figure 3C,D), this was diminished in mice of the *Isolated* and *Malformed/Dead* litter groups (Figures 4I and S7I). To investigate whether KYN and KA levels at E9.5 were lowest in mothers with the most severely affected litters, we stratified the *Malformed/Dead* group into subgroups according to malformation load (average number of defects within the litter). Despite low replicate numbers, KYN and KA showed a trend of inverse correlation with malformation load at E9.5 (Figures 4J and S7J).

Concentrations of NAD Salvage Pathway excretion metabolites 1MNA, 2PY, and 4PY are considered to reflect overall NAD availability because they are only produced under conditions of surplus NAM [43, 44, 45]. Though levels of these metabolites were generally low in mothers on *Limited Diet*, there were significant differences in their concentration between litter phenotype categories (Figures 4C and S7A, Table S6) and matching trends in the malformation load subgroups (Figures 4D and S7B). 1MNA levels were detectable at all post‐implantation stages (≥ E6.5) and increased between E9.5 and E12.5 in mothers of the *Normal* group (Figure 4C, Table S5). Similarly, 4PY levels were detectable in this group after E9.5, and levels increased between E9.5 and E12.5 (Figure S7A,B, Table S5). Mothers of the *Isolated* group had lower levels of both excretion metabolites at E6.5 than the *Normal* group but showed a similar increase between E9.5 and E12.5 (Figures 4C,D and S7A,B, Table S5). By contrast, mice of the *Malformed/Dead* litter group had even lower 1MNA and 4PY levels, often below the detection limit, from E6.5 to E12.5 (Figures 4C,D and S7A,B, Table S5).

Maternal NAD^+^ levels differed between the litter phenotype categories over time, with the difference significant at E12.5 (*p* = 0.0019; Table S6, Figure 4E). NAD^+^ levels also showed a trend of inverse correlation with malformation load (Figure 4F). The Salvage Pathway metabolites and vitamin B3 vitamers NAM and NMN showed the same inverse correlation with malformation load at E12.5 (Figure S7D,F). Further, although there was an upward trend in NAD^+^ levels in the *Normal* and *Isolated* groups from E9.5 onward, levels declined between E9.5 and E12.5 in the *Malformed/Dead* group (Figure 4E). This inverted concentration course from E9.5 to E12.5 between *Normal/Isolated* and *Malformed/Dead* groups was also observed when the NAD Salvage Pathway metabolites (NAD^+^, NAM, and NMN) were combined, and this was significant at E12.5 (Figure 4G, Table S6). The combined data for NAD^+^, NAM, and NMN show that the observed differences of NAD^+^ levels at E12.5 in mice with a high malformation load are not due to a shift from NAD^+^ to NAM. Instead, these mice had a decline in all metabolites of the Salvage Pathway. Combined with observations for 1MNA, 2PY, and 4PY, these data show that overall NAD availability in maternal blood declined in mothers with affected litters, particularly between E9.5 and E12.5, and that those with the strongest decline had the most severe malformation phenotypes in their litters.

## 4 Discussion

Embryonic NAD levels at critical stages of development are highly variable between litters on the same NAD precursor restricted diet, but correlate with maternal NAD metabolite levels in the circulation [17, 19] because they are dependent on maternal NAD precursor provision. Thus, the formation and type of defect(s) that arise likely depend on both the timing, extent, and duration of NAD deficiency during organogenesis [21]. Yet, this theory relies on snapshot measurements of maternal and embryonic metabolites taken at defined endpoints. These can determine maternal and embryonic levels at the time of organ development, but this will preclude any knowledge of the defect types the embryo would have presented later. Or NAD levels can be determined at the time of CNDD phenotypic presentation but then cannot be linked back to NAD metabolome alterations at earlier stages that triggered the formation of defects. Such snapshot assessments are especially limited in their ability to elucidate defect causation in the context of gene–environment interactions because environmental drivers of defect formation, unlike genetic factors, may occur only transiently during gestation.

To address this, we tracked the maternal NAD metabolome and embryonic volume longitudinally using UHPLC–MS/MS and MRI. We combined this with detailed phenotyping to temporally link maternal NAD precursor provision and embryo growth during organogenesis with embryo phenotype at a later developmental stage. This study represents the first time that the maternal NAD metabolome has been longitudinally measured in the same mouse over gestation. As observed previously [19, 21], maternal levels of the Salvage Pathway excretion metabolites, 1MNA, 2PY, and 4PY, increase throughout gestation under *Standard Diet* conditions. KYN levels also dynamically and reproducibly peak in the maternal whole blood at E9.5 and then decline, similar to previous observations in the serum shown to reflect placental IDO/TDO2 expression [27].

Of particular importance is the vitamin B3 vitamer NAM. It is the major circulatory NAD precursor for cells, including those of the embryo, to generate NAD via the Salvage Pathway [20] and its maternal circulatory levels correlate with embryonic NAD synthesis [19]. Normal embryos were generated on both diets and grew comparably, as observed previously [21], indicating that maternal NAD precursor provision was sufficient. Indeed, NAM as well as NMN levels were not different in mothers with normal litters on either diet. Yet, the *Limited Diet* induced other significant changes in the maternal NAD metabolome. The most notable metabolic consequence of *Limited Diet* provision was a sustained reduction in all Salvage Pathway excretion products 1MNA, 2PY and 4PY to low nanomolar levels, as observed previously [8, 19, 21]. These metabolites are generated when NAM levels are in excess to prevent feedback inhibition of NAD‐consuming enzymes [43, 44, 45, 46]. Conversely, a lowering or lack of 1MNA, 2PY and 4PY reflects a universal decline in NAM and NAD availability and conservation of NAM levels to maintain Salvage Pathway activity. A recent clinical study found that elevated circulatory and urinary levels of 1MNA, 2PY and 4PY were associated with a higher risk of recurrent miscarriage, suggesting that NAD salvage pathway activity and excretion product generation need to be regulated to prevent adverse pregnancy outcomes [9].

Despite the overall reduction in excretion metabolite levels on the *Limited Diet*, mothers with entirely normal litters had higher levels at all post‐implantation (>E6.5) stages of development than those with affected litters. Since NAD^+^ levels were also stable throughout, it is likely that NAD^+^ levels in mothers with normal embryos exceeded both their own and their embryos' requirements. The increase in excretion metabolite levels between E9.5 and E12.5 in mothers with normal litters on either diet indicates an increase in NAD precursor provision during this critical window of organogenesis, aligning with the essentiality of NAD in regulating the rate of normal development [47]. Combined, the NAD metabolic profiles of mothers with normal litters on the *Limited Diet* show their embryos had sufficient NAD precursors to develop normally. Although unmonitored, it is highly likely that these females on the *Limited Diet* consumed more of the TRP‐containing water during pregnancy, enabling them to maintain circulatory NAD precursor levels throughout the critical stages of pregnancy. Conversely, mothers with malformed/dead embryos likely consumed less water, resulting in insufficient provision of NAD precursors to their embryos during organogenesis.

Embryos that were malformed at E15.5 or died in utero showed evidence of this fate by mid‐gestation. Malformed embryos were significantly smaller than their normal counterparts by E9.5, and resorbed embryos were, on average, smaller still. Most of these deaths occurred early, between E9.5 and E12.5. By contrast, given the high incidence of defects in their littermates, the four embryos that died between E12.5 and E15.5 were possibly lost because their defects were incompatible with life or due to placental deficits. However, placental defects were not detected in malformed embryos of another CNDD mouse model [21]. Regardless of how many embryos in a litter went on to develop defects, the adverse effects of the *Limited Diet* on maternal NAD precursor provision were observable at E6.5. Although maternal NAD^+^ levels did not differ, all affected litters had lower NAD precursor availability as evidenced by very low or absent 1MNA levels at E6.5. At subsequent stages E9.5 and E12.5, NAD^+^, NAM and NMN showed an inverse correlation with defect load, confirming that reduced NAD precursor availability was the metabolic consequence of *Limited Diet* treatment, and this was consistent with reduced growth by E9.5. However, the size at E9.5 neither reflected the number, type, nor combination of defects that developed in that embryo. Further studies are required to elucidate the molecular mechanisms that disrupt embryogenesis in an NAD‐dependent manner during mid‐gestation to cause malformation, and whether, for example, perturbation of DNA repair, mitochondrial biogenesis, chromatin remodeling or NAD‐dependent shifts in glucose metabolism during gastrulation [48].

Malformed embryos showed a varied spectrum of defects between litters, from isolated to multiple CNDD‐associated defects. Defect combinations were not random and instead co‐occurring defect patterns emerged within, but not between, litters. In a few litters, not all embryos had defects. In these litters, embryos may have been at slightly different developmental stages and thus had different NAD precursor demands at specific phases of organogenesis, causing some embryos to be affected, whereas others developed normally. Indeed, we observed some variability among embryo volumes within litters at E9.5, E12.5 and at E15.5. Furthermore, embryos with a high malformation load, that is, those with many malformed organs/tissues, also had more severe defect types within organs, as exemplified by the shift from kidney hypoplasia and microphthalmia to kidney agenesis and anophthalmia in embryos with higher overall defect load. Higher numbers of defects also correlated with less growth between E9.5 and E12.5, corresponding to organogenesis, demonstrating that most defects form between these stages. In those embryos with only kidney and lung hypoplasia, growth was not affected, likely because hypoplasia is caused by reduced progenitor cell proliferation after E11.5/E12.5 [49, 50].

These findings also indicate that malformation occurrence is driven by the timing and duration of NAD deficiency, resulting from specific temporal windows of insufficient maternal NAD precursor supply. Though phenotypes and the fluctuation in NAD precursor provision that caused them are unique to each pregnancy, mothers can be grouped by litter outcome. Blood levels of NAD^+^ and its Salvage Pathway precursor metabolites NAM and NMN were lower during organogenesis (E9.5–12.5) in mice that had litters of entirely malformed or dead embryos. The inverse correlation between maternal NAD^+^, NAM, and NMN levels and malformation load was strongest at E12.5. Insufficient Salvage Pathway metabolite availability and little to no detection of NAM‐derived excretion metabolites have been observed in previous dietary NAD precursor restriction mouse models linked to CNDD [8, 19, 21]. Such a decline in Salvage Pathway activity in the maternal blood cells likely affected the maternal ability to release sufficient NAD precursors, primarily NAM, into the circulation for the developing embryos to generate NAD during organogenesis and thus resulted in malformation.

By contrast, in mothers with normal or mildly affected litters, levels of these metabolites increased from E9.5 to E12.5, thereby supporting normal development of most embryos. At E12.5, maternal circulatory levels of NAD^+^ and all Salvage Pathway metabolites combined declined as malformation load increased, indicating that the likelihood of defects in organs developing during organogenesis is proportional to the decline in NAD precursor availability between E9.5 and E12.5. By contrast, lung and kidney hypoplasia likely stemmed from insufficient NAD precursor availability at or near E12.5, but these embryos did not develop other defects because earlier maternal NAD precursor supply was sufficient for proper organ development in these cases. Further, the two smallest embryos in these litters had additional defects beyond lung and kidney, likely because they were developmentally delayed, which possibly resulted in a relatively earlier onset of NAD deficiency, affecting additional organs. It has to be noted that although the timing of NAD deficiency during embryogenesis explains variability in phenotypes among the CNDD spectrum, it is unknown why the development of other organs/tissues is not affected by NAD deficiency to an extent that visible structural anomalies arise.

In summary, this study determined that malformation occurrence and severity are directly linked to maternal NAD precursor provision and that the timing of insufficient provision, and therefore NAD deficiency, determines defect type. Several NAD‐related metabolites spanning both the NAD *de novo* Synthesis Pathway (KYN, KA) and the NAD Salvage Pathway (NAD excretion metabolites: 1MNA, 2PY, 4PY) are reliable indicators of murine maternal NAD status. Unlike such well‐controlled inbred mouse models, numerous other factors are expected to contribute to the risk of NAD deficiency in the human clinical context [4]. In addition to dietary factors, a range of genetic modifiers and maternal pathophysiological conditions may modulate NAD precursor availability in the fetus [51, 52, 53, 54, 55]. Given that the metabolites identified here are generally reflective of maternal NAD status, they may have general utility as biomarkers for the early prediction of embryonic NAD precursor availability and risk of CNDD‐related adverse pregnancy outcome, irrespective of whether the cause of NAD deficiency is genetic, environmental, or due to a combination of factors.

## Author Contributions

K.B., H.C., and S.L.D. conceived the study; K.B., H.C., N.S., G.C., and S.L.D. designed the study; K.B., H.C., N.S., E.M.M.A.M., G.C., and D.Z.S. performed research and acquired data; K.B., H.C., E.M.M.A.M., G.C., and D.Z.S. analyzed data; S.L.D. acquired financial support; K.B., H.C., G.C., and S.L.D. drafted the manuscript; all authors contributed to data interpretation and revised the manuscript.

## Funding

This work was supported by DHAC | National Health and Medical Research Council (NHMRC), ID1135886, ID2007896, ID1162878. NSW Health Cardiovascular Research Capacity Program. New South Wales Government (nswgov). Freedman Foundation. New South Wales Cardiovascular Research Network—VCCRI Research Innovation Grant.

## Conflicts of Interest

The authors declare no conflicts of interest.

## Supporting information


**Figure S1:** Representative litter on the *Standard Diet* at E9.5, showing each embryo tracked and segmented.
**Figure S2:** Representative litter on the *Standard Diet* at E12.5, showing each embryo tracked and segmented.
**Figure S3:** Isoflurane treatment at E6.5 affects embryo volume but has no effect on embryo malformation phenotype.
**Figure S4:** Embryo volumes show variability between litters and within litters.
**Figure S5:** Specific organ/tissue defect subsets co‐occur at E15.5.
**Figure S6:** Maternal blood metabolite levels at E0.5 show variability, but this is independent of the phenotypic litter outcome later in gestation.
**Figure S7:** Pregnant females with affected litters had NAD metabolomic differences compared to females with unaffected litters.
**Table S1:** Overview of mouse diets.
**Table S2:** Embryo volume by phenotype over gestation.
**Table S3:** Whole blood NAD metabolite levels in pregnant mice on *Standard Diet* and pregnant mice on *Limited Diet* with normal litters (litters without malformation).
**Table S4:** Whole blood NAD metabolite levels in pregnant mice at the end of pre‐treatment (E0.5), categorized by litter phenotypic outcome.
**Table S5:** Whole blood NAD metabolite levels in pregnant mice, sorted by litter phenotypic outcome.
**Table S6:** One‐way ANOVA using the Kruskal‐Wallis test for maternal whole blood NAD metabolite levels on the *Limited Diet* by litter category.

## Data Availability

All data generated or analyzed during this study are included in this article and its Supporting Information files.
