# Supplementary material for: Timing of NAD Deficiency During Organogenesis Dictates Defect Type and Penetrance
Source: FASEB J. 2026 Feb 3;40(3):e71504. doi: 10.1096/fj.202502824RRR (PMC12865519; doi:10.1096/fj.202502824RRR)
Supplement: Supplementary file 1 — Figure S1: Representative litter on the Standard Diet at E9.5, showing each embryo tracked and segmented. Figure S2: Representative litter on the Standard Diet at E12.5, showing each embryo tracked and segmented. Figure S3: Isoflurane treatment at E6.5 affects embryo volume but has no effect on embryo malformation phenotype. Figure S4: Embryo volumes show variability between litters and within litters. Figure S5: Specific organ/tissue defect subsets co‐occur at E15.5. Figure S6: Maternal blood metabolite levels at E0.5 show variability, but this is independent of the phenotypic litter outcome later in gestation. Figure S7: Pregnant females with affected litters had NAD metabolomic differences compared to females with unaffected litters. Table S1: Overview of mouse diets. Table S2: Embryo volume by phenotype over gestation. Table S3: Whole blood NAD metabolite levels in pregnant mice on Standard Diet and pregnant mice on Limited Diet with normal litters (litters without malformation). Table S4: Whole blood NAD metabolite levels in pregnant mice at the end of pre‐treatment (E0.5), categorized by litter phenotypic outcome. Table S5: Whole blood NAD metabolite levels in pregnant mice, sorted by litter phenotypic outcome. Table S6: One‐way ANOVA using the Kruskal‐Wallis test for maternal whole blood NAD metabolite levels on the Limited Diet by litter category. [file FSB2-40-e71504-s001.zip › fsb271504-sup-0001-FigureS1.docx]

**Supplementary Information for**

Timing of NAD deficiency during organogenesis dictates defect type and penetrance

Kayleigh Bozon^1,2^, Hartmut Cuny^1,3,*^, Nana Sunn^4^, Ella MMA Martin^1,2^, Delicia Z. Sheng^1,2^, Gavin Chapman^1,3^, Sally L. Dunwoodie^1,3,*^

^1^Developmental and Stem Cell Biology Division, Victor Chang Cardiac Research Institute, Sydney, NSW 2010, Australia.

^2^University of New South Wales, Sydney, New South Wales, Australia

^3^School of Clinical Medicine, Faculty of Medicine and Health, University of New South Wales, Sydney, NSW 2052, Australia.

^4^Innovation Centre, Victor Chang Cardiac Research Institute, Sydney, NSW 2010, Australia.

***Corresponding authors:**

Sally L. Dunwoodie **Email:** [s.dunwoodie@victorchang.edu.au](mailto:s.dunwoodie@victorchang.edu.au),

Hartmut Cuny **Email:** [h.cuny@victorchang.edu.au](mailto:h.cuny@victorchang.edu.au)

Victor Chang Cardiac Research Institute, 405 Liverpool Street, Darlinghurst NSW 2010, Australia

P: +61 2 9295 8613

**Table of contents**

| **Content** | **Page** |
| --- | --- |
| **Supplemental Figures** | **3** |
| **Figure S1.** Representative litter on the *Standard Diet* at E9.5, showing each embryo tracked and segmented. | 3 |
| **Figure S2.** Representative litter on the *Standard Diet* at E12.5, showing each embryo tracked and segmented. | 4 |
| **Figure S3:** Isoflurane treatment at E6.5 affects embryo volume but has no effect on embryo malformation phenotype. | 5 |
| **Figure S4:** Embryo volumes show variability between litters and within litters. | 6 |
| **Figure S5:** Specific organ/tissue defect subsets co-occur at E15.5. | 7 |
| **Figure S6:** Maternal blood metabolite levels at E0.5 show variability, but this is independent of the phenotypic litter outcome later in gestation. | 8 |
| **Figure S7:** Pregnant females with affected litters had NAD metabolomic differences compared to females with unaffected litters. | 9 |
| **Supplemental Tables** | **10** |
| **Table S1.** Overview of mouse diets | 10 |
| **Table S2.** Embryo volume by phenotype over gestation | 11 |
| **Table S3.** Whole blood NAD metabolite levels in pregnant mice on *Standard Diet* and pregnant mice on *Limited Diet* with normal litters (litters without malformation) | 12 |
| **Table S4.** Whole blood NAD metabolite levels in pregnant mice at the end of pre-treatment (E0.5), categorized by litter phenotypic outcome. | 13 |
| **Table S5.** Whole blood NAD metabolite levels in pregnant mice, sorted by litter phenotypic outcome. | 14 |
| **Table S6.** One-way ANOVA using the Kruskal-Wallis test for maternal whole blood NAD metabolite levels on the *Limited Diet* by litter category | 15 |


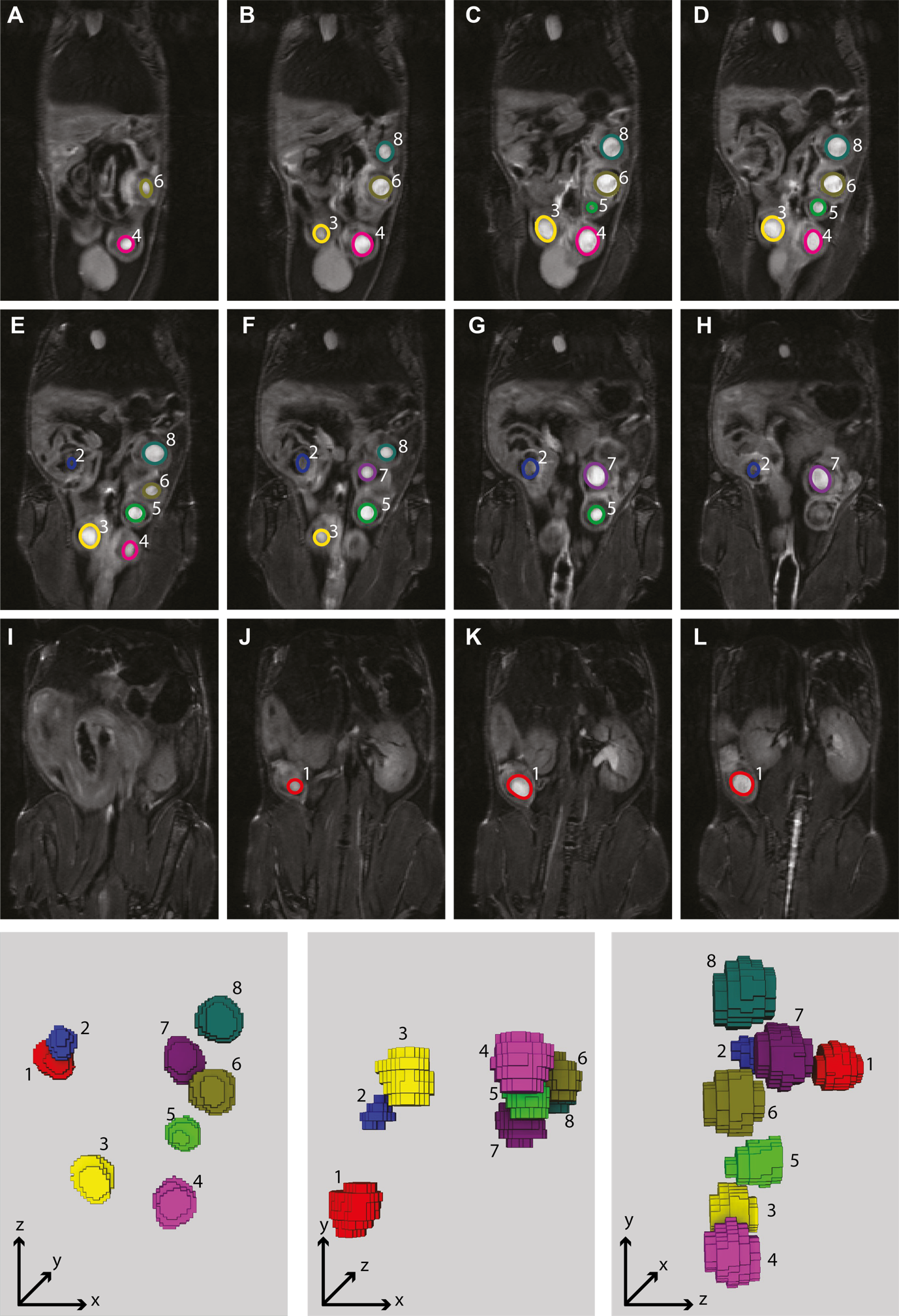


**Figure S1. Representative litter on the *Standard Diet* at E9.5, showing each embryo tracked and segmented.** (A–L) Sequential slices from ventral to dorsal through the mouse. Bottom panels show a 3D volume rendering of all embryos within the litter in x, y and z orientation.


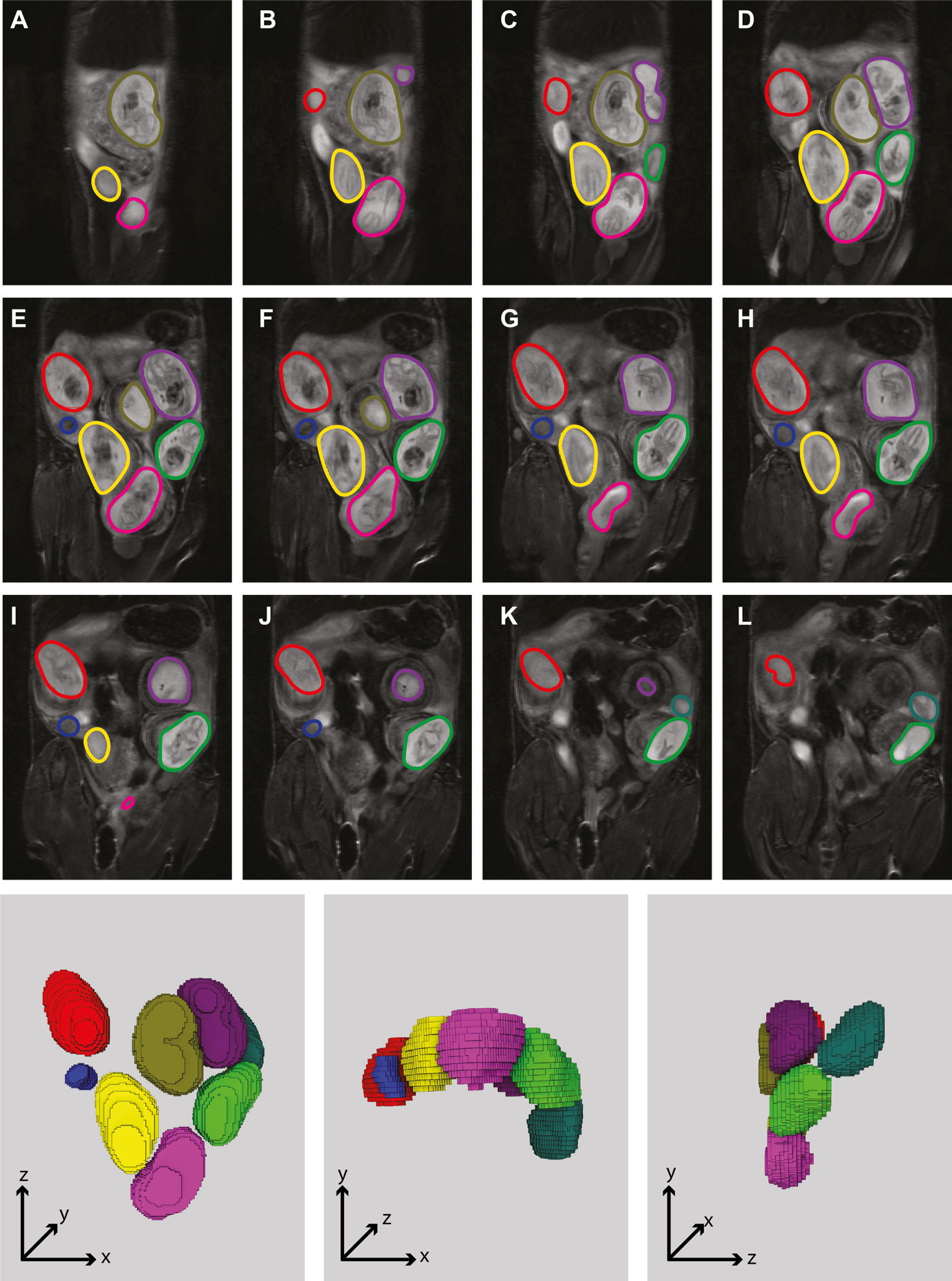


**Figure S2. Representative litter on the *Standard Diet* at E12.5, showing each embryo tracked and segmented.** (A–L) Sequential slices from ventral to dorsal through the mouse. Bottom panels show a 3D volume rendering of all embryos within the litter in x, y and z orientation.


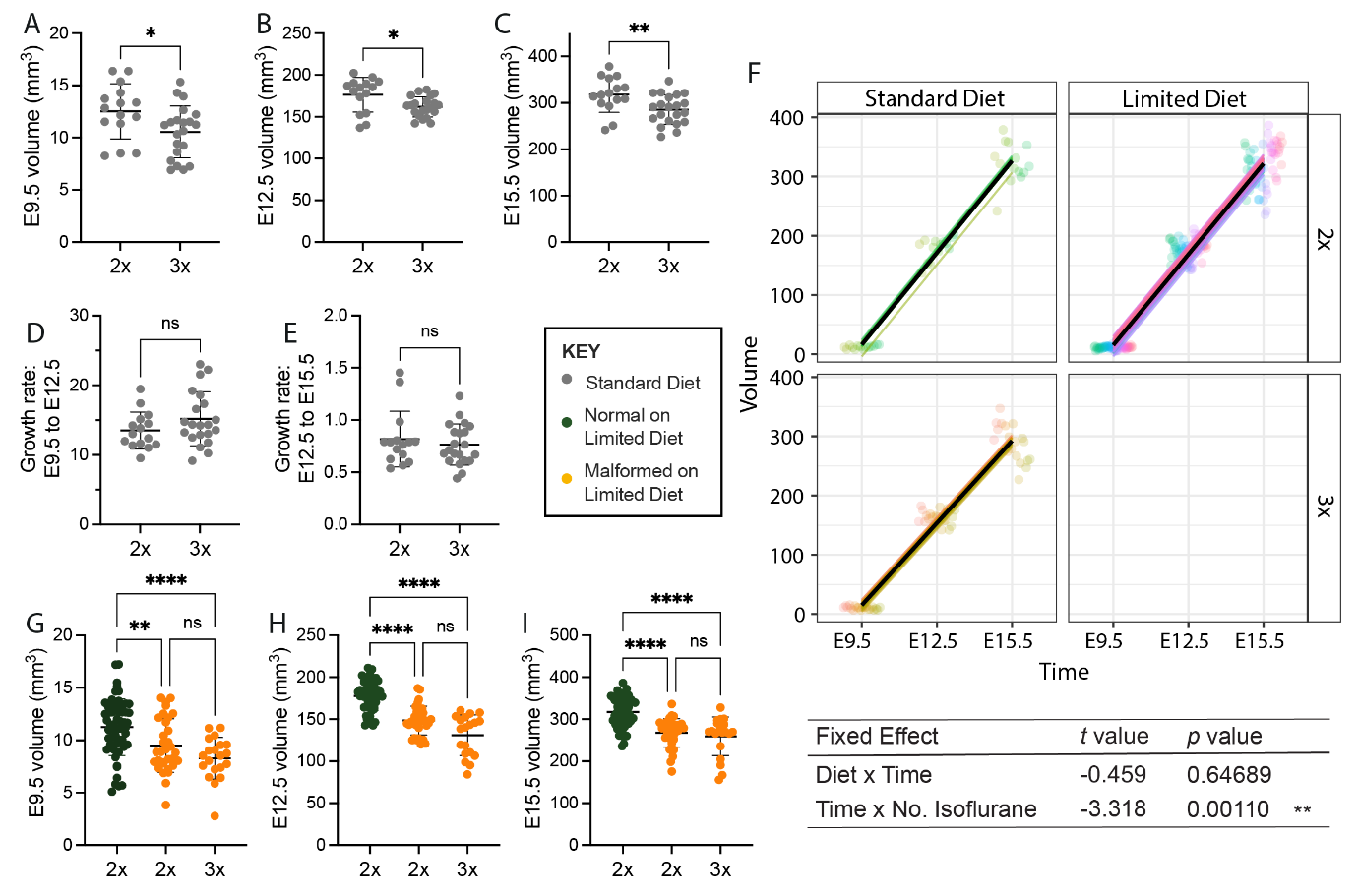


**Figure S3: Isoflurane treatment at E6.5 affects embryo volume but has no effect on embryo malformation phenotype**. (A-C) Comparison of the volumes of embryos from pregnant females on *Standard Diet* that had been anaesthetized using isoflurane and MRI imaged at two timepoints (E9.5, E12.5 = 2× treatment) with those treated at three timepoints (E6.5, E9.5, E12.5 = 3× treatment) at E9.5 (A), E12.5 (B), and E15.5 (C). (D, E) Comparison of embryo growth rates between the 2× and 3× isoflurane-treated pregnant females. (D) Growth rate between E9.5 and E12.5, defined as volume at E12.5 – E9.5 divided by volume at E9.5. (E) Growth rate between E12.5 and E15.5, defined as volume at E15.5 – E12.5 divided by volume at E12.5. (F) Linear mixed model analyses generated in RStudio. Only embryos without malformation were used for this analysis. The resulting linear mixed model was visualized using the flexplot R package. Black line represents the fitted linear mixed model per group. Colored dots represent individual embryo volumes and lines represent random effect slopes. Statistical calculation outputs are shown in the table below the graphs. (G-I) Comparison of volumes at E9.5 (G), E12.5 (H), and E15.5 (I) of embryos from mothers on *Standard* or *Limited Diet*. Embryos were grouped according to the number of isoflurane treatments (2× or 3×) and embryo phenotype (normal or malformed) irrespective of diet. Each dot in (A-E) and (G-I) represents an embryo and bars indicate mean±standard deviation. Significance of difference between groups in (A-E) was assessed by unpaired t-test and in (G-I) by Kruskal-Wallis one-way ANOVA with Dunn’s multiple comparisons test (*****p*<0.0001, ***p*<0.01, **p*<0.05; ns, not significant).


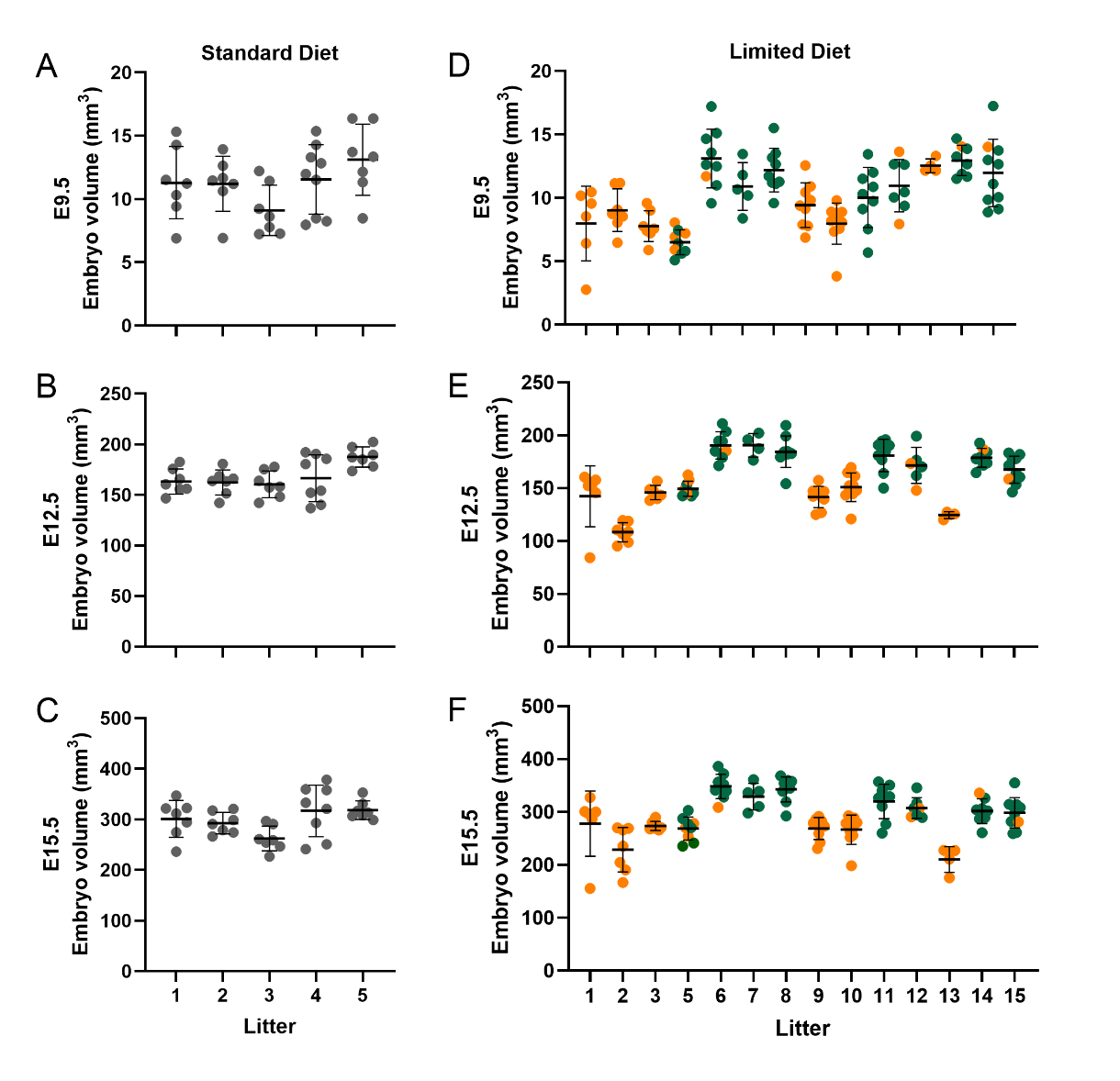


**Figure S4:** **Embryo volumes show variability between litters and within litters.** (A-C) Embryo volumes at E9.5 (A), E12.5 (B), and E15.5 (C) of the *Standard Diet* group, separated by litters. (D-F) Embryo volumes at E9.5 (D), E12.5 (E), and E15.5 (F) of the *Limited Diet* group, separated by litters. Embryos that died *in utero* are not shown; this includes litter 4 of the *Limited Diet* group in which all embryos resorbed. Each dot represents an embryo, and bars indicate mean±standard deviation. Colors indicate whether an embryo in the *Limited Diet* group was phenotypically normal (green) or malformed (orange). Litter numbers correspond to Figure 1C.


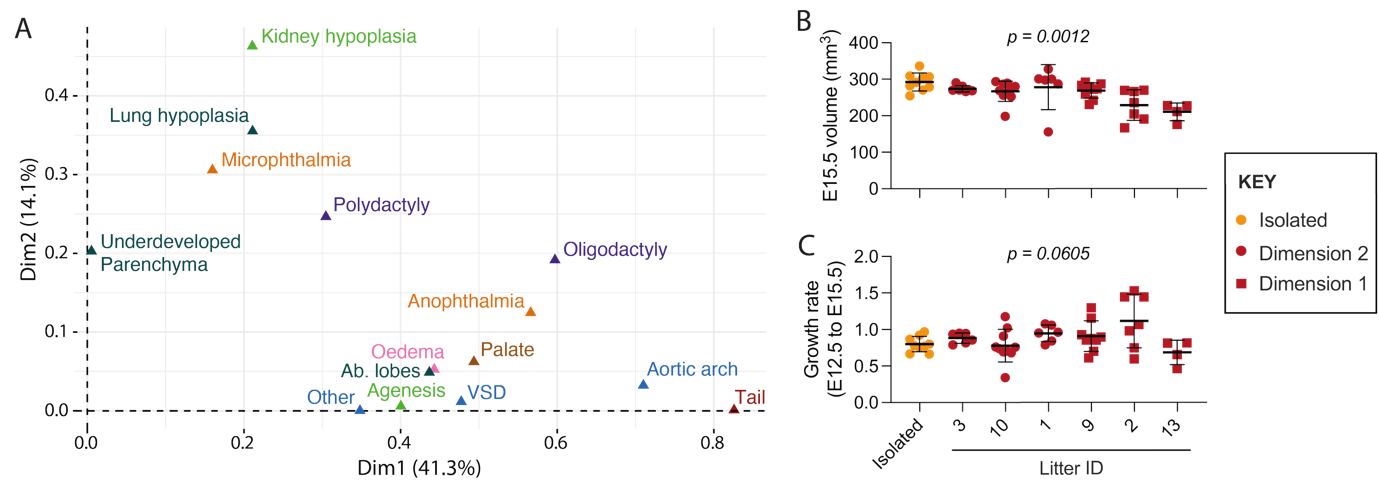


**Figure S5: Specific organ/tissue defect subsets co-occur at E15.5.** (A) Multiple correspondence analysis (MCA) to assess co-occurrences of defect subsets. Defect subset categories were removed from analyses if there were insufficient incidences (n <5) amongst *Limited Diet* embryos. Colors match those used for main organ/tissue categories in Figure 2A. All defects except underdeveloped parenchyma also aligned with Dimension 1. (B-C) Comparison of embryo volumes at E15.5 (B) and the growth rate from E12.5 to E15.5 (C) between litters with defects in <1 embryo per litter (litters 15, 6, 14, 5 and 12 grouped and categorized as *Isolated*) and those litters with an average of >1 defect per embryo (litters 3, 10, 1, 8, 2, 13; each litter shown individually). Litters 3, 10 and 1 align with MCA Dimension 2 (circles), whilst litters 9, 2 and 13 align with MCA Dimension 1 (squares). Each dot represents an embryo, and bars indicate mean±standard deviation. Statistical difference between the groups was determined by Kruskal-Wallis one-way ANOVA.


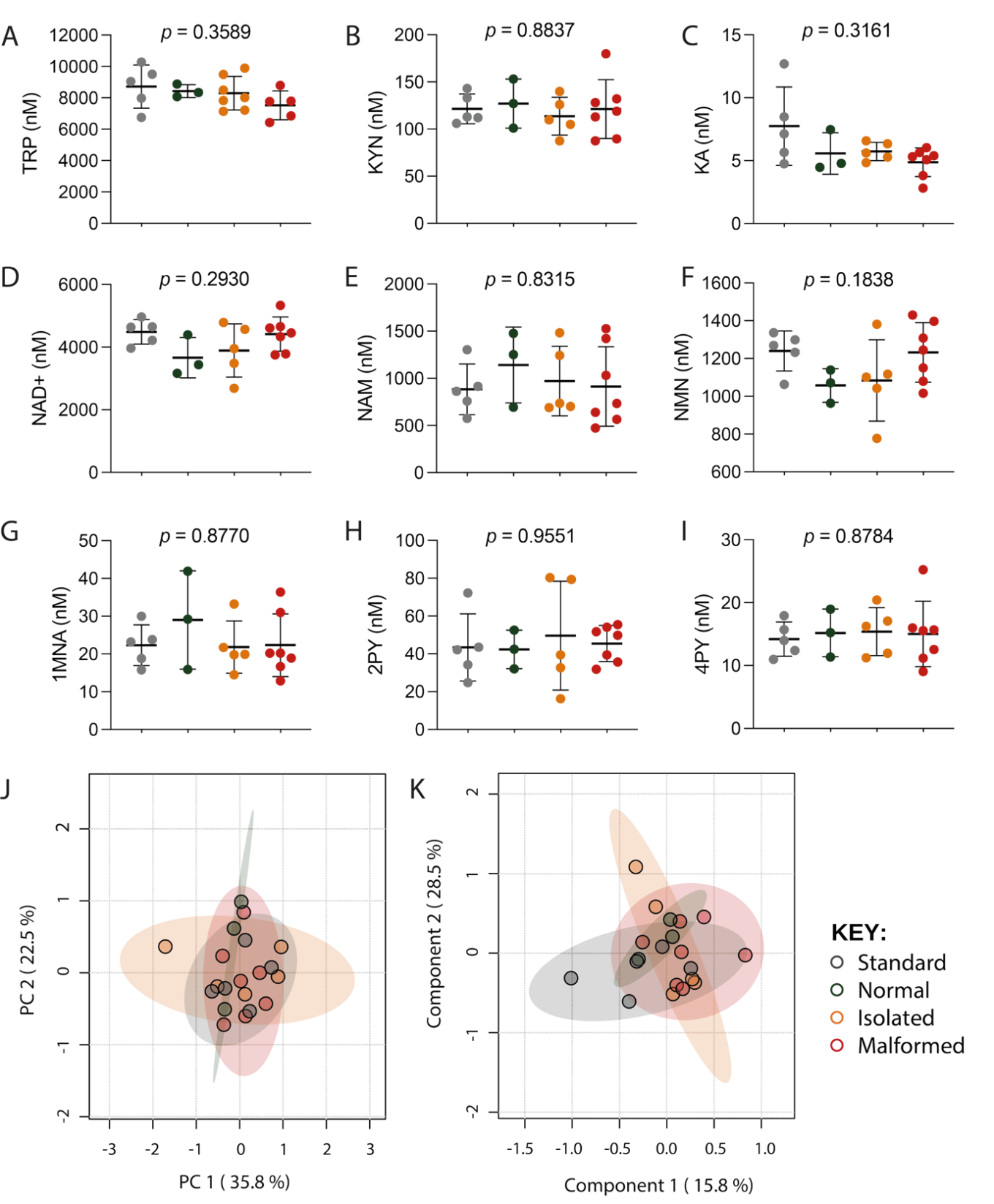


**Figure S6:** **Maternal blood metabolite levels at E0.5 show variability, but this is independent of the phenotypic litter outcome later in gestation.** (A-I) Metabolite levels measured at E0.5 in whole blood of mothers on the *Standard Diet* (grey) and mothers on *Limited Diet*, separated by the malformation load of their litters: *Normal* (none of the embryos of the litter had malformations; green), *Isolated* (average number of affected organs/tissues <1 per embryo; orange), and *Malformed/Dead* (average number of affected organs/tissues >1 per embryo; red). Each dot represents a blood sample, and bars indicate mean±standard deviation. The *p* values from Kruskal-Wallis one-way ANOVA are indicated above each graph and also provided in Table S4. (J) Principal Component Analysis of metabolite levels including both diets and all categories as in (A-I). Concentration values were standardized (log10 transformed and Pareto scaled) for analysis. (K) Partial Least Squares Discriminant Analysis (PLS-DA) of the same values as in (J). TRP, tryptophan; KYN, kynurenine; KA, kynurenic acid; NAD^+^, nicotinamide adenine dinucleotide (oxidized); NAM, nicotinamide; NMN, nicotinamide mononucleotide; 1MNA, 1-methylnicotinamide; 2PY, N-methyl-2-pyridone-5-carboxamide; 4PY, N-methyl-4-pyridone-3-carboxamide.


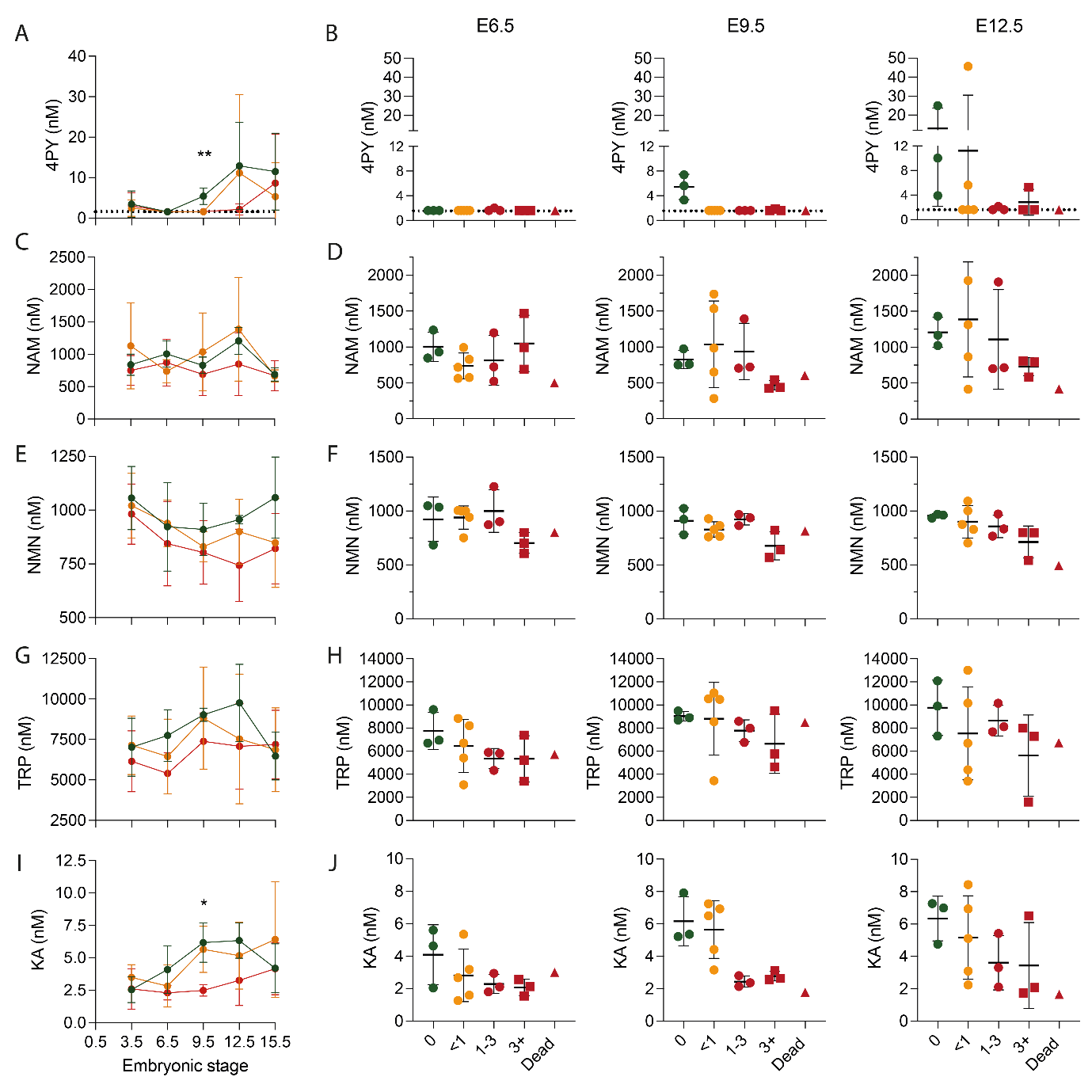


**Figure S7:** **Pregnant females with affected litters had NAD metabolomic differences compared to females with unaffected litters.** (A, C, E, G, I) Blood metabolite levels longitudinally measured from E3.5 to E15.5 in mothers on the *Limited Diet*. Colours indicate the groups based on malformation load of their litters: *Normal* (all embryos of the litter unaffected; green), *Isolated* (average number of affected organs/tissues <1 per embryo; orange), and *Malformed/Dead* (average number of affected organs/tissues >1 per embryo; red). Significantly different values between the groups at a given timepoint, determined by Kruskal-Wallis one-way ANOVA are indicated (***p*<0.01, **p*<0.05). See Table S6 for numerical *p* values. (B, D, F, H, J) Scatter plots of the values at E6.5, E9.5, and E12.5 of the respective metabolites in (A, C, E, G, I). Each dot represents a blood sample and bars indicate mean±standard deviation. The Malformed/Dead group was stratified into three subgroups (average number of affected organs/tissues 1-3 (circles), >3 (squares), or all embryos dead (triangles) to highlight trends between malformation load and metabolite levels. The detection limit of 4PY is outlined by a dotted line. 4PY, N-methyl-4-pyridone-3-carboxamide; NAM, nicotinamide; NMN, nicotinamide mononucleotide; TRP, tryptophan; KA, kynurenic acid.

**Table S1. Overview of mouse diets**

| **Name** | **Feed code^†^** | **Vitamin B3 in feed (mg/kg)** | **TRP in feed (mg/kg)** | **TRP in water (mg/L)** | **Dietary TRP (mg/d)** | **Dietary NAD precursors (µg/d)** |
| --- | --- | --- | --- | --- | --- | --- |
| Standard Diet | SF22-101 | 30 | 2000 | - | 7.8 | 247 |
| Limited Diet | SF21-083 | 0 | 0 | 600 | 3.72 | 62 |

^†^Product code of the manufacturer (Specialty Feeds, Glen Forrest, Australia).

TRP, tryptophan.

**Table S2. Embryo volume by phenotype over gestation**

| **Diet** | **Embryo Phenotype** | **E9.5** | | | **E12.5** | | | **E15.5** | | |
| --- | --- | --- | --- | --- | --- | --- | --- | --- | --- | --- |
|  |  | **Volume** (mm^3^) | ***n*** | ***p*** | **Volume**  (mm^3^) | ***n*^†^** | ***p*** | **Volume**  (mm^3^) | ***n*** | ***p*** |
| Standard | Standard | 11.37 ± 2.70 | 36 | >0.9999 | 168.0 ± 17.5 | 36 | 0.3368 | 298.9 ± 37.6 | 36 | 0.0699 |
| Limited | Normal | 11.26 ± 2.71 | 57 |  | 177.6 ± 17.4 | 54 |  | 317.4 ± 35.2 | 36 |  |
| Limited | Malformed | 9.03 ± 2.41 | 52 | 0.0004 | 141.9 ± 21.6 | 51 | <0.0001 | 264.4 ± 38.7 | 52 | <0.0001 |
| Limited | Dead | 6.94 ± 3.57 | 22 | <0.0001 | 39.06 ± 49.23 | 22 | <0.0001 | - | - | - |

^†^At E12.5, there were embryos where part of the conceptus was outside the boundary of the MRI images/slices. Therefore, number of embryos per stage (*n*) reflects the number of conceptuses where a full volumetric measurement could be taken for that stage.

Volume values represent the mean±standard deviation. The *p* values were calculated by Kruskal-Wallis one-way ANOVA with Dunn’s multiple comparisons test for each embryo phenotype category compared to Normal embryos on the *Limited Diet*.

**Table S3. Whole blood NAD metabolite levels in pregnant mice on *Standard Diet* and pregnant mice on *Limited Diet* with normal litters (litters without malformation)**

|  | **Standard Diet** (n = 5) | | | | | |  |  | **Normal Litters on Limited Diet** (n = 3) | | | | | |  |
| --- | --- | --- | --- | --- | --- | --- | --- | --- | --- | --- | --- | --- | --- | --- | --- |
|  | **E0.5** | **E3.5** | **E6.5** | **E9.5** | **E12.5** | **E15.5** | ***p*** |  | **E0.5** | **E3.5** | **E6.5** | **E9.5** | **E12.5** | **E15.5** | ***p*** |
| TRP  (µM) | 8.71  ±  1.38 | 9.30 ± 1.21 | 8.83 ± 1.31 | 10.05 ± 1.89 | 9.32  ±  1.32 | 8.82  ±  0.91 | 0.548 |  | 8.43  ±  0.41 | 7.02 ± 1.79 | 7.75 ± 1.60 | 9.02 ± 0.41 | 9.76  ±  2.39 | 6.48  ±  1.48 | 0.257 |
| KYN  (nM) | 121.4  ±  16.0 | 76.1 ± 11.8 | 229.4 ± 38.1 | 509.1 ± 169.8 | 227.8  ±  33.0 | 210.0  ±  15.4 | **0.004** |  | 127.2  ±  26.0 | 57.4 ± 27.6 | 161.5 ± 36.1 | 307.2 ± 100.1 | 186.9  ±  58.7 | 130.4 ±  5.1 | 0.050 |
| KA  (nM) | 7.75 ± 3.13 | 5.76 ± 0.91 | 4.68 ± 1.18 | 8.40 ± 2.13 | 6.55 ± 0.89 | 6.56 ± 2.26 | 0.118 |  | 5.58 ± 1.65 | 2.55 ± 1.00 | 4.10 ± 1.84 | 6.17 ± 1.52 | 6.34 ± 1.37 | 4.20 ± 1.88 | 0.177 |
| NAM  (µM) | 0.88 ± 0.27 | 1.04 ± 0.32 | 0.92 ± 0.47 | 0.81 ± 0.19 | 0.90 ± 0.19 | 1.00 ± 0.35 | 0.680 |  | 1.14 ± 0.40 | 0.84 ± 0.16 | 1.00 ± 0.20 | 0.83 ± 0.13 | 1.21 ± 0.21 | 0.69 ± 0.10 | 0.208 |
| NAD^+^ (µM) | 4.49 ± 0.39 | 4.18 ± 0.92 | 4.32 ± 0.61 | 4.30 ± 0.80 | 4.31 ± 0.58 | 4.42 ± 1.29 | 0.901 |  | 3.66 ± 0.65 | 3.59 ± 0.11 | 3.19 ± 0.92 | 3.34 ± 0.61 | 3.63 ± 0.19 | 3.67 ± 0.40 | 0.637 |
| NMN  (µM) | 1.24 ± 0.11 | 1.04 ± 0.14 | 1.07 ± 0.18 | 1.07 ± 0.16 | 1.02 ± 0.13 | 1.02 ± 0.24 | 0.269 |  | 1.06 ± 0.09 | 1.06 ± 0.15 | 0.92 ± 0.21 | 0.91 ± 0.12 | 0.96 ± 0.02 | 1.06 ± 0.19 | 0.436 |
| 1MNA (nM) | 22.34 ± 5.42 | 33.83 ± 6.92 | 29.57 ± 15.19 | 34.61 ± 5.68 | 49.35 ± 5.68 | 50.34 ± 17.44 | **0.024** |  | 29.03 ± 12.99 | 3.11 ± 1.67 | 4.53 ± 1.29 | 3.67 ± 1.12 | 8.72 ± 4.79 | 10.38 ± 4.67 | 0.061 |
| 2PY  (nM) | 43.39 ± 17.73 | 58.29 ± 17.76 | 48.95 ± 13.22 | 48.12 ± 17.33 | 83.57 ± 21.34 | 55.30 ± 14.99 | **0.017** |  | 42.39 ± 10.20 | 6.25 ± 0.00 | 6.25 ± 0.00 | 6.25 ± 0.00 | 17.70 ± 13.47 | 13.08 ± 11.84 | **0.037** |
| 4PY  (nM) | 33.90 ± 12.50 | 46.84 ± 17.36 | 38.31 ± 6.07 | 40.90 ± 16.99 | 64.99 ± 12.41 | 39.39 ± 8.62 | **0.018** |  | 30.33 ± 4.42 | 3.46 ± 3.28 | 1.56 ± 0.00 | 5.42 ± 2.04 | 12.90 ± 10.79 | 11.49 ± 9.50 | 0.053 |

Values represent the mean±standard deviation. Metabolite levels below the limit of detection (LOD) were given a value LOD/2 to allow statistical comparisons. The LOD was 3.125 nM for 1MNA, 12.5 nM for 2PY, and 3.125 nM for 4PY. Significance of difference across timepoints within the same diet group was assessed by Kruskal-Wallis one-way ANOVA.

TRP, tryptophan; KYN, kynurenine; KA, kynurenic acid; NAM, nicotinamide, NAD^+^, nicotinamide adenine dinucleotide (oxidized); NMN, nicotinamide mononucleotide; 1MNA, 1-methylnicotinamide; 2PY, N-methyl-2-pyridone-5-carboxamide; 4PY, N-methyl-4-pyridone-3-carboxamide.

**Table S4. Whole blood NAD metabolite levels in pregnant mice at the end of pre-treatment (E0.5), categorized by litter phenotypic outcome.**

|  | **Standard Diet^†^** | **Limited Diet** | | | **One-way ANOVA (*p*)** | **CoV** |
| --- | --- | --- | --- | --- | --- | --- |
|  |  | **Normal^‡^** | **Isolated^§^** | **Malformed/Dead^¶^** |  |  |
| TRP (µM) | 8.71 ± 1.38 | 8.43 ± 0.41 | 7.52 ± 0.92 | 8.29 ± 1.07 | 0.2656 | 12.73 |
| KYN (nM) | 121.40 ± 15.79 | 127.00 ± 26.00 | 113.64 ± 20.13 | 121.29 ± 31.18 | 0.8231 | 18.75 |
| KA (nM) | 7.74 ± 3.11 | 5.57 ± 1.65 | 5.73 ± 0.73 | 4.87 ± 1.14 | 0.2451 | 33.62 |
| NAM (µM) | 0.88 ± 0.27 | 1.14 ± 0.40 | 0.97 ± 0.37 | 0.91 ± 0.42 | 0.8449 | 36.05 |
| NAD^+^ (µM) | 4.49 ± 0.39 | 3.66 ± 0.65 | 3.89 ± 0.85 | 4.42 ± 0.54 | 0.2163 | 15.27 |
| NMN (µM) | 1.24 ± 0.11 | 1.06 ± 0.09 | 1.08 ± 0.22 | 1.23 ± 0.16 | 0.2368 | 13.80 |
| 1MNA (nM) | 22.32 ± 5.40 | 29.00 ± 13.00 | 22.32 ± 6.91 | 22.32 ± 8.31 | 0.8831 | 33.11 |
| 2PY (nM) | 43.38 ± 17.75 | 42.37 ± 10.20 | 49.64 ± 28.84 | 45.44 ± 9.57 | 0.9722 | 36.37 |
| 4PY (nM) | 33.92 ± 12.49 | 30.30 ± 4.43 | 37.62 ± 20.89 | 33.42 ± 6.64 | 0.9511 | 34.56 |

^†^Litters on *Standard Diet* (n = 5)

^‡^Normal litters on *Limited Diet* (n = 3)

^§^Average number of affected organs/tissues <1 per embryo (n = 5)

^¶^Average number of affected organs/tissues >1 per embryo (n = 7)

Values represent the mean±standard deviation. The *p* values were calculated by Kruskal-Wallis one-way ANOVA.

TRP, tryptophan; KYN, kynurenine; KA, kynurenic acid; NAM, nicotinamide, NAD^+^, nicotinamide adenine dinucleotide (oxidized); NMN, nicotinamide mononucleotide; 1MNA, 1-methylnicotinamide; 2PY, N-methyl-2-pyridone-5-carboxamide; 4PY, N-methyl-4-pyridone-3-carboxamide; CoV, coefficient of variation.

**Table S5. Whole blood NAD metabolite levels in pregnant mice, sorted by litter phenotypic outcome.**

|  |  | **Normal** (n = 3)^†^ | | | | |  | **Isolated** (n = 5)^‡^ | | | | |  | **Malformed/Dead** (n = 7)^§^ | | | | |
| --- | --- | --- | --- | --- | --- | --- | --- | --- | --- | --- | --- | --- | --- | --- | --- | --- | --- | --- |
|  |  | **E3.5** | **E6.5** | **E9.5** | **E12.5** | **E15.5** |  | **E3.5** | **E6.5** | **E9.5** | **E12.5** | **E15.5** |  | **E3.5** | **E6.5** | **E9.5** | **E12.5** | **E15.5** |
| TRP (µM) |  | 7.02 ± 1.79 | 7.75 ± 1.60 | 9.02 ± 0.41 | 9.76 ± 2.39 | 6.48 ± 1.48 |  | 7.14 ± 1.82 | 6.45 ± 2.31 | 8.81 ± 3.15 | 7.53 ± 4.01 | 6.87 ± 2.59 |  | 6.15 ± 1.89 | 5.40 ± 1.27 | 7.39 ± 1.74 | 7.08 ± 2.65 | 7.19 ± 2.12 |
| KYN (nM) |  | 57.4 ± 27.6 | 161.5 ± 36.1 | 307.2 ± 100.1 | 186.9 ± 58.7 | 130.4 ± 5.1 |  | 58.1 ± 22.6 | 83.4 ± 55.1 | 249.5 ± 127.8 | 166.5 ± 65.1 | 183.6 ± 50.5 |  | 49.6 ± 25.4 | 92.2 ± 38.7 | 160.6 ± 55.5 | 161.0 ± 75.3 | 167.5 ± 57.5 |
| KA (nM) |  | 2.55 ± 1.00 | 4.10 ± 1.84 | 6.17 ± 1.52 | 6.34 ± 1.37 | 4.20 ± 1.88 |  | 3.49 ± 0.99 | 2.82 ± 1.62 | 5.65 ± 1.78 | 5.17 ± 2.58 | 6.42 ± 4.46 |  | 2.61 ± 1.56 | 2.31 ± 0.56 | 2.49 ± 0.44 | 3.26 ± 1.95 | 4.16 ± 2.00 |
| NAM (µM) |  | 0.84 ± 0.16 | 1.00 ± 0.20 | 0.83 ± 0.13 | 1.21 ± 0.21 | 0.69 ± 0.10 |  | 1.13 ± 0.66 | 0.74 ± 0.18 | 1.04 ± 0.60 | 1.39 ± 0.80 | 0.66 ± 0.10 |  | 0.75 ± 0.23 | 0.87 ± 0.36 | 0.69 ± 0.33 | 0.85 ± 0.49 | 0.67 ± 0.23 |
| NAD^+^ (µM) |  | 3.59 ± 0.11 | 3.19 ± 0.92 | 3.34 ± 0.61 | 3.63 ± 0.19 | 3.67 ± 0.40 |  | 3.68 ± 0.77 | 3.87 ± 0.21 | 2.95 ± 0.50 | 3.09 ± 0.34 | 3.59 ± 0.46 |  | 4.04 ± 0.58 | 3.09 ± 0.57 | 2.95 ± 0.43 | 2.55 ± 0.43 | 2.86 ± 0.84 |
| NMN (µM) |  | 1.06 ± 0.15 | 0.92 ± 0.21 | 0.91 ± 0.12 | 0.96 ± 0.02 | 1.06 ± 0.19 |  | 1.02 ± 0.15 | 0.94 ± 0.11 | 0.83 ± 0.07 | 0.90 ± 0.15 | 0.85 ± 0.21 |  | 0.98 ± 0.14 | 0.84 ± 0.20 | 0.80 ± 0.15 | 0.74 ± 0.17 | 0.82 ± 0.16 |
| 1MNA (nM) |  | 3.11 ± 1.67 | 4.53 ± 1.29 | 3.67 ± 1.12 | 8.72 ± 4.79 | 10.38 ± 4.67 |  | 2.81 ± 1.73 | 1.78 ± 0.49 | 3.16 ± 1.63 | 10.01 ± 14.68 | 9.98 ± 13.07 |  | 2.26 ± 1.26 | 1.56 ± 0.00 | 1.72 ± 0.42 | 2.04 ± 1.27 | 8.25 ± 11.49 |
| 2PY (nM) |  | 6.25 ± 0.00 | 6.25 ± 0.00 | 6.25 ± 0.00 | 17.70 ± 13.47 | 13.08 ± 11.84 |  | 6.25 ± 0.00 | 6.25 ± 0.00 | 6.25 ± 0.00 | 16.06 ± 21.95 | 10.43 ± 9.35 |  | 6.90 ± 1.71 | 6.25 ± 0.00 | 6.25 ± 0.00 | 6.25 ± 0.00 | 14.73 ± 14.94 |
| 4PY (nM) |  | 3.46 ± 3.28 | 1.56 ± 0.00 | 5.42 ± 2.04 | 12.90 ± 10.79 | 11.49 ± 9.50 |  | 2.47 ± 2.02 | 1.56 ± 0.00 | 1.56 ± 0.00 | 11.18 ± 19.35 | 5.32 ± 8.41 |  | 3.03 ± 3.30 | 1.63 ± 0.17 | 1.60 ± 0.10 | 2.17 ± 1.38 | 8.65 ± 12.15 |

^†^Normal litters on *Limited Diet*

^‡^Average number of affected organs/tissues <1 per embryo

^§^Average number of affected organs/tissues >1 per embryo

Values represent the mean±standard deviation. Metabolite levels below the limit of detection (LOD) were given a value LOD/2 to allow statistical comparisons. The LOD was 3.125 nM for 1MNA, 12.5 nM for 2PY, and 3.125 nM for 4PY.

TRP, tryptophan; KYN, kynurenine; KA, kynurenic acid; NAM, nicotinamide, NAD^+^, nicotinamide adenine dinucleotide (oxidized); NMN, nicotinamide mononucleotide; 1MNA, 1-methylnicotinamide; 2PY, N-methyl-2-pyridone-5-carboxamide; 4PY, N-methyl-4-pyridone-3-carboxamide.

**Table S6. One-way ANOVA using the Kruskal-Wallis test for maternal whole blood NAD metabolite levels on the *Limited Diet* by litter category**

|  | **E3.5** | **E6.5** | **E9.5** | **E12.5** | **E15.5** |
| --- | --- | --- | --- | --- | --- |
| TRP | 0.5883 | 0.1552 | 0.1929 | 0.5462 | 0.8863 |
| KYN | 0.9105 | 0.1259 | 0.1028 | 0.9154 | 0.1583 |
| KA | 0.3877 | 0.4330 | **0.0004** | 0.0848 | 0.6372 |
| NAM | 0.6668 | 0.4181 | 0.2816 | 0.1865 | 0.9154 |
| NAD^+^ | 0.5127 | 0.1929 | 0.6303 | **0.0019** | 0.1461 |
| NMN | 0.7175 | 0.4664 | 0.6204 | 0.1351 | 0.1726 |
| 1MNA | 0.6416 | **0.0009** | **0.0232** | **0.0051** | 0.4642 |
| 4PY | 0.8846 | >0.9999 | **0.0022** | 0.0807 | 0.2038 |
| NAD^+^, NAM, NMN combined | 0.7638 | 0.3158 | 0.3871 | **0.0480** | 0.1216 |

Statistical analyses refer to the data shown in Figure 4C, E, G, I and Figure S7A, C, G, I. One-way ANOVA was not performed for N-methyl-2-pyridone-5-carboxamide (2PY) because levels were below the limit of quantification for most samples at all timepoints.

TRP, tryptophan; KYN, kynurenine; KA, kynurenic acid; NAM, nicotinamide, NAD^+^, nicotinamide adenine dinucleotide (oxidized); NMN, nicotinamide mononucleotide; 1MNA, 1-methylnicotinamide; 4PY, N-methyl-4-pyridone-3-carboxamide.
